# Identification of genes required for *Plasmodium* gametocyte-to-sporozoite development in the mosquito vector

**DOI:** 10.1016/j.chom.2023.08.010

**Published:** 2023-09-13

**Authors:** Chiamaka Valerie Ukegbu, Ana Rita Gomes, Maria Giorgalli, Melina Campos, Alexander J. Bailey, Tanguy Rene Balthazar Besson, Oliver Billker, Dina Vlachou, George K. Christophides

**Affiliations:** 1 Department of Life Sciences, Imperial College London, London SW7 2AZ, UK; 2 Wellcome Trust Sanger Institute, Wellcome Trust Genome Campus, Hinxton CB10 1SA, UK

## Abstract

Malaria remains one of the most devastating infectious diseases. Reverse genetic screens offer a powerful approach to identify genes and molecular processes governing malaria parasite biology. However, the complex regulation of gene expression and genotype-phenotype associations in the mosquito vector, along with sexual reproduction, have hindered the development of screens in this critical part of the parasite life cycle. To address this, we developed a genetic approach in the rodent parasite *Plasmodium berghei* that, in combination with barcode sequencing, circumvents the fertilization roadblock and enables screening for game-tocyte-expressed genes required for parasite infection of the mosquito *Anopheles coluzzii*. Our results confirm previous findings, validating our approach for scaling up, and identify genes necessary for mosquito midgut infection, oocyst development, and salivary gland infection. These findings can aid efforts to study malaria transmission biology and to develop interventions for controlling disease transmission.

## Graphical abstract

**Graphical abstract:**
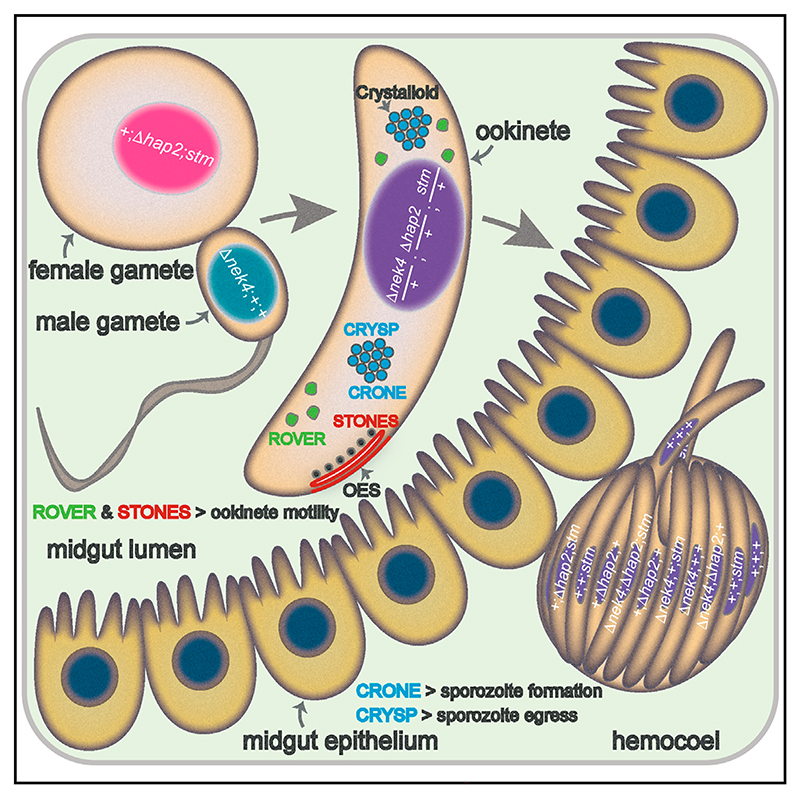

## Introduction

Enhanced vector control and improved health care have reduced malaria cases and deaths. However, mosquito resistance to insecticides and changes in mosquito behavior have limited the impact of these measures. Furthermore, the only licensed antimalarial vaccine (RTS, S) is not expected to have a universal game-changing impact, highlighting the need for additional tools targeting disease transmission.

Malaria is caused by the protozoan parasite *Plasmodium*, transmitted to humans by *Anopheles* mosquitoes. Transmission begins when a female mosquito ingests haploid *Plasmodium* gametocytes that transform inside the mosquito midgut into gametes and then zygotes. While undergoing meiosis, a diploid and then tetraploid zygote becomes motile ookinete that traverses the midgut epithelium. Most ookinetes are eliminated by the mosquito immune system during this process. In the midgut sub-epithelial space, the ookinete transforms into the replicative oocyst where thousands of haploid sporozoites develop over approximately 2 weeks through endomitotic replication and budding. Following egress from the oocyst, the sporozoites migrate to the salivary glands, ready to be transmitted to another human upon a next mosquito bite.

The relationship between gene expression and protein function in *Plasmodium* development is complex. Within the mosquito, parasite development coincides with notable changes in transcriptome repertoires,^1,2^ facilitated by transcription factors of the Apetala 2 (AP2) family.^3,4^ Posttranscriptional regulation is also important,^5,6^ as seen in the synthesis and storage of transcripts in the female gametocyte, released for translation after fertilization.^7–9^ The AP2-O transcription factor controls *de novo* gene expression in the zygote and ookinete, and some proteins produced in ookinetes are transported via the crystalloid, an organelle exclusive to ookinetes and young oocysts, to function during oocyst development.^10^

The spatiotemporal mismatch between gene expression and protein function, together with increased ploidy in the zygote and ookinete and endomitosis in the oocyst, has hindered the development of genetic screens to study mosquito infection and disease transmission. Efficient tools combining the scalability of signature-tagged mutagenesis (STM) and throughput of barcode sequencing have enabled high throughput genetic screens in haploid asexual blood stages (ABSs) of the rodent parasite *Plasmodium berghei* (*P. berghei*).^11,12^ However, cross-fertilization between mutants in the mosquito blood bolus limits the utility of barcoded mutants for identifying gene functions before products of meiosis and endomitotic replication cycles segregate during sporogony,^13^ necessitating more elaborate genetic designs.

Here, we present a reverse genetics screen design that enables the study of genes involved in the gametocyte-to-sporozoite development. Guided by our earlier discovery that the male *P. berghei* genome is largely inactive in the first 32 h in the mosquito,^2^ our approach involves STM in female gametes that are crossed to wild-type (WT) male gametes, leading to zygotes that carry female null and male WT alleles. Apart from preventing barcodes from being transmitted through male gametes, this approach precludes the generation of double mutants that could reduce the analytical power of the screen when co-inherited mutations have strong and/or interacting phenotypes. We first validated the design by confirming phenotypes of previously studied genes and concluded that this is a powerful strategy for scaling up the rate at which functions could be assigned to genes transcriptionally enriched in gametocytes. We then characterized three such unstudied genes identified by the screen, all encoding putative transmembrane proteins, and found that *STONES* encodes a protein associated with the ookinete extrados site (OES) and required for ookinete motility, while *CRYSP* and *CRONE* encode crystalloid proteins required for sporozoite formation and oocyst egress and/or salivary gland infection, respectively. We also investigated two genes not detected by the screen but identified in our published and unpublished data as important for transmission. We confirmed that *PIMMS57*, which we previously showed to encode a protein required for oocyst development,^14^ and *ROVER*, which encodes a previously uncharacterized protein essential for ookinete motility associated with vesicle trafficking, have knockout phenotypes that are fully rescued by the male WT alleles. This is consistent with the predicted shortcoming of the screen to reveal recessive phenotypes in diploid cells, further proving the validity of our design for discovering genes and processes important for *Plasmodium* transmission biology.

## Results and Discussion

### Identification of gametocyte-enriched transcripts *in vivo*

**Figure 1:**
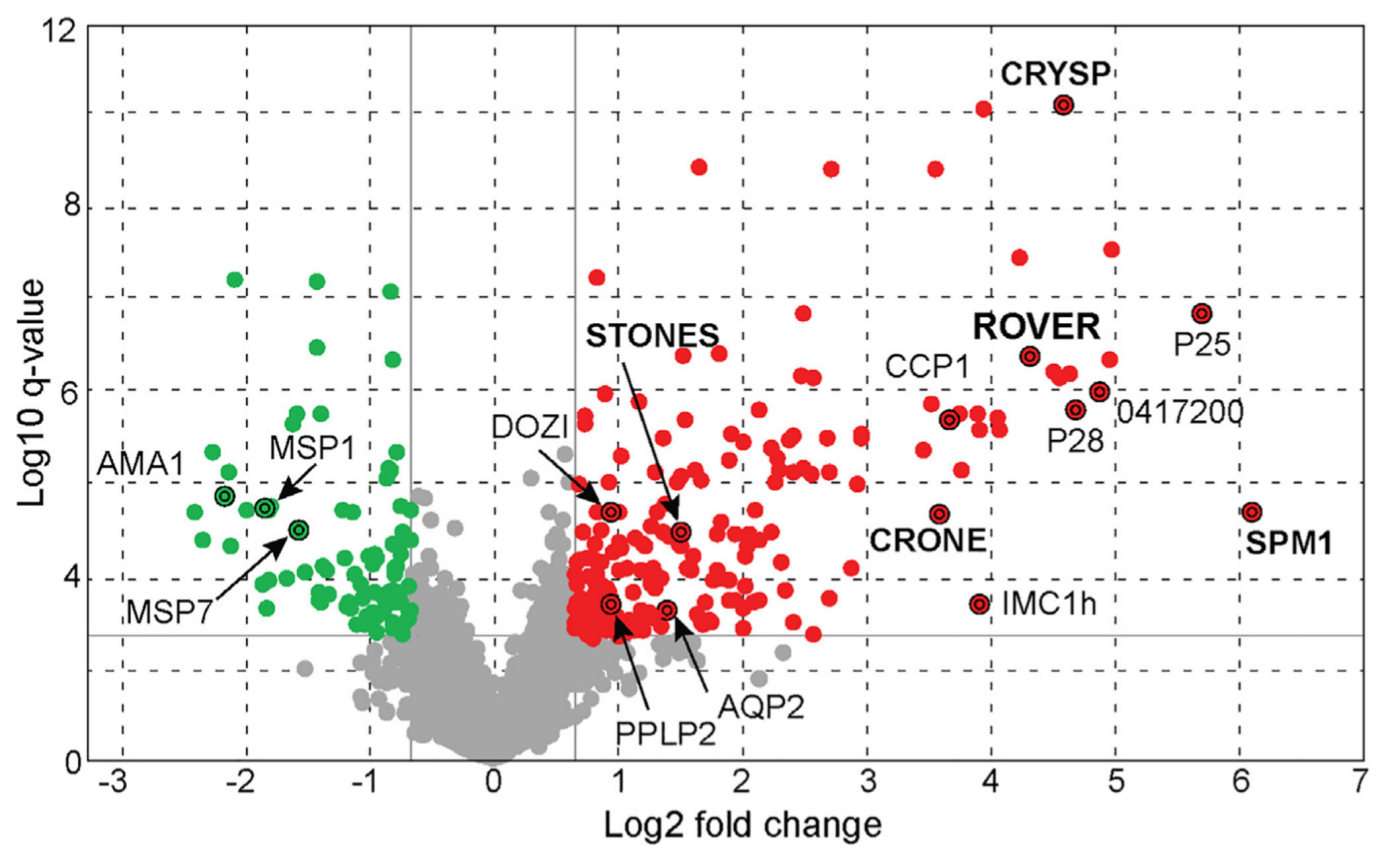
Transcriptional enrichment in *P. berghei* gametocytes Volcano plot showing differential gene expression measurements between the ANKA 2.34 WT and ANKA 2.33 non-gametocyte-producing *P. berghei* lines 1 hpi in the *A. coluzzii* midgut. Red and green dots represent the 274 genes that are regulated by at least 1.6-fold, respectively, in the ANKA 2.34 WT line compared with the ANKA 2.33 line. Gray dots represent genes that do not show significant regulation. 189 genes (red dots) were enriched in the ANKA 2.34 compared with the ANKA 2.33 line and include genes involved in sexual and sporogonic development such as *PPLP2, DOZI, P25, P28, IMC1h*, and *LAP2*. Eighty-five genes (green) were identified to be downregulated and include genes encoding putative blood stage proteins such as *AMA1* and the merozoite surface proteins *MSP1* and *MSP7*. Genes in bold are those selected for further characterization.

To identify gametocyte-enriched genes in the mosquito midgut, we infected *Anopheles coluzzii* (*A. coluzzii*) (previously *Anopheles gambiae* [*A. gambiae*] M form) with *P. berghei* ANKA 2.34 or ANKA 2.33 (non-gametocyte-producing) lines. RNA isolated from the mosquito midguts 1 h post blood feeding (pbf) was analyzed using a *P. berghei* oligonucleotide microarray.^1^ Three replicate infections were performed that allowed us to examine the expression of 3,428 genes, after excluding probes ambiguously mapping to the genome. We identified 189 transcripts with significant >1.6-fold enrichment in ANKA 2.34 compared with ANKA 2.33 (Figure 1; Table S1). Among these, 109 were previously shown to be affected in parasites lacking the DEAD-box RNA helicase DOZI (development of zygote inhibited), which is essential for translational mRNA repression in female gametocytes^9^; DOZI was among these 109 genes. Comparison with single-cell RNA sequencing of gametocytes^15^ revealed that 74% of the upregulated genes (139 genes) are highly expressed in gametocytes with 83% of them (116 genes) being enriched in female gametocytes.

### STM screen optimization

We generated a library of barcoded knockout *Plasmo*GEM vectors targeting gametocyte-enriched genes, as well as control genes of known or no phenotypes. In this initial study, we pooled 27 vectors targeting 13 previously characterized genes (including PBANKA_0720900), 6 genes encoding 6-cys domain proteins, and 5 uncharacterized genes detected in our transcriptomic study, which are translationally repressed by DOZI^9^: PBANKA_0413500 (*STONES*), PBANKA_1338100 (*CRYSP*), PBANKA_1353800 (*ROVER*), PBANKA_0810700 (*SPM1*), and PBANKA_0720900 (*CRONE*). The pool also contained 4 epitope-tagging *Plasmo*GEM vectors with no fitness cost in ABS development.^11,12^ Thus, the total number of *Plasmo*GEM vectors in the pool was 31.

**Figure 2:**
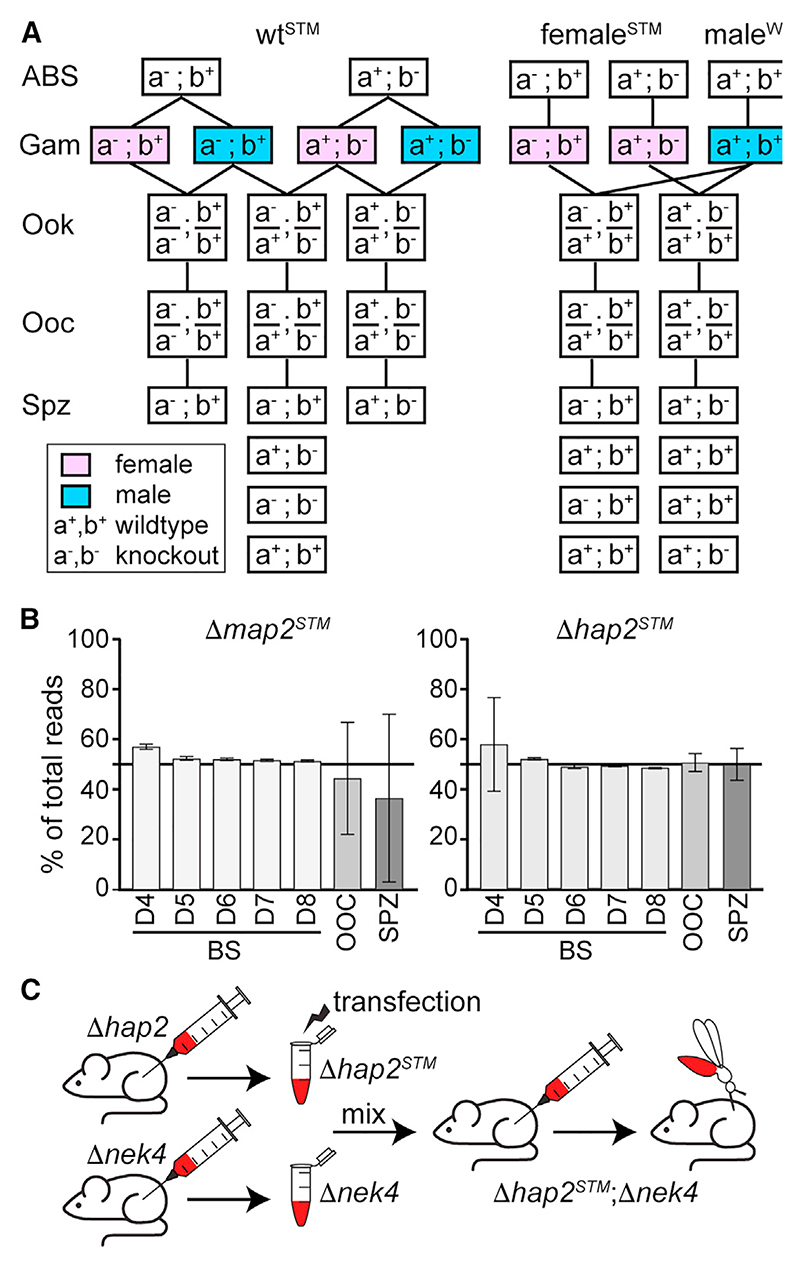
STM genetic and experimental design (A) Parasite genotypes after mutagenesis in WT parasites (WT^STM^) or female-donor line (female^STM^, e.g., *Δmap2*^*STM*^ and *Δhap2*^*STM*^) crossed to WT male-donor line (male^WT^, e.g., *Δnek4*^*WT*^). Example of two STM mutant loci (a and b) shown. (B) Growth dynamics of *Δmap2* and *Δhap2* in mouse blood stages (BSs) 4–8 days post transfection with STM pool and in oocyst and salivary gland sporozoites after crossing to *Δnek4* during *A. coluzzii* infection. Percentage of map2 and hap2 barcode counts in total barcode counts shown. Whiskers show SEM. (C) Experimental approach schematic with mutagenesis carried out in *Δhap2* (*Δhap2*^*STM*^), mixed with *Δnek4* to infect mice, and followed by mosquito infections with derived transheterozygous parasites. ABSs, asexual blood stages; BSs, blood stages; Gam, gametes; Ook, ookinetes; Ooc, oocysts; Spz, sporozoites.

STM in WT *P. berghei* has been shown to be effective in ABS screens,^11,12^ but when viable mutants were transmitted through the life cycle, the power to detect phenotypes specific to one sex or during the diploid phase of the life cycle was limited until products of meiosis had segregated during sporogony.^13^ We sought to overcome the latter by mutagenesis in a female-donor line that would be then crossed to a male-only donor line, leading to unidirectional crosses and no double mutants (Figure 2A). In this design, complementation by the WT male alleles is addressed by a previous finding that the male genome is largely silent during the first 32 h after fertilization,^2^ except for genes specifically expressed in the zygote/ookinete. Thus, our design allows for the screening of phenotypes in mosquito stages associated with gene expression in the female gametocyte, mostly caused by maternal transcript or protein deposition, or with constitutive gene expression.

We evaluated two potential female-donor lines: *Δmap2* and *Δhap2*. The absence of the mitogen-activated protein kinase microtubule-associated protein (MAP2) hampers male gamete exflagellation by affecting cytokinesis and axoneme motility,^16–18^ while the absence of the male-specific membrane fusogen HAP2 renders the male gametes unable to fuse with the female gametes.^19–21^ To generate selection marker-free *Δmap2* and *Δhap2* lines, we used barcoded *Plasmo*GEM vectors and a negative selection strategy^22^ (Figure S1A). Integration of the disruption cassettes was confirmed by PCR. After transfection of *Δmap2* or *Δhap2* with the pool of barcoded vectors, each line was used to infect mice together with the *Δnek4* line that lacks viable female gametes.^23,24^ This co-infection leads to fertilization of *Δmap2*^*STM*^ or *Δhap2*^*STM*^ female gametes by *Δnek4* male gametes that do not carry any other mutations. Blood samples were collected from the infected mice at days 4–8 post infection (pi), while mosquito midguts and salivary glands were sampled at days 12 and 21 pbf. Consequently, each parasite was expected to carry two barcodes: one identifying the genetic background (*Δmap2* or *Δhap2*), accounting for approximately 50% of the total barcode counts, and another from the 31-vector pool. Analyzing the abundance of each barcode and its changes over time allowed us to track the growth dynamics of the mutant populations.

The results demonstrated that starting from day 5 after mouse infection, once drug selection had eliminated parasites with no STM vector integration, the barcode counts of both *map2* or *hap2* reached a stable level of approximately 50% of the total count, as expected, with very little variability between replicates (Figure 2B). However, in oocysts and salivary gland sporozoites, the *map2* barcodes were highly variable and less than 50% of the total counts. This observation indicated that the *Δmap2* line may have defective sporozoite development and would not be suitable for our screen. Consequently, we proceeded with the *Δhap2* female-donor line for further experimentation. Therefore, our chosen approach involved introducing STM mutations in *Δhap2* parasites (*Δhap2*^*STM*^), which then co-infected mice alongside *Δnek4* parasites, followed by mosquito infections (Figure 2C). Mutagenesis in WT parasites served the purpose of assessing fitness and stability and acted as reference for the mosquito infections.

### Fitness and stability of mutants in the mouse host

**Figure 3:**
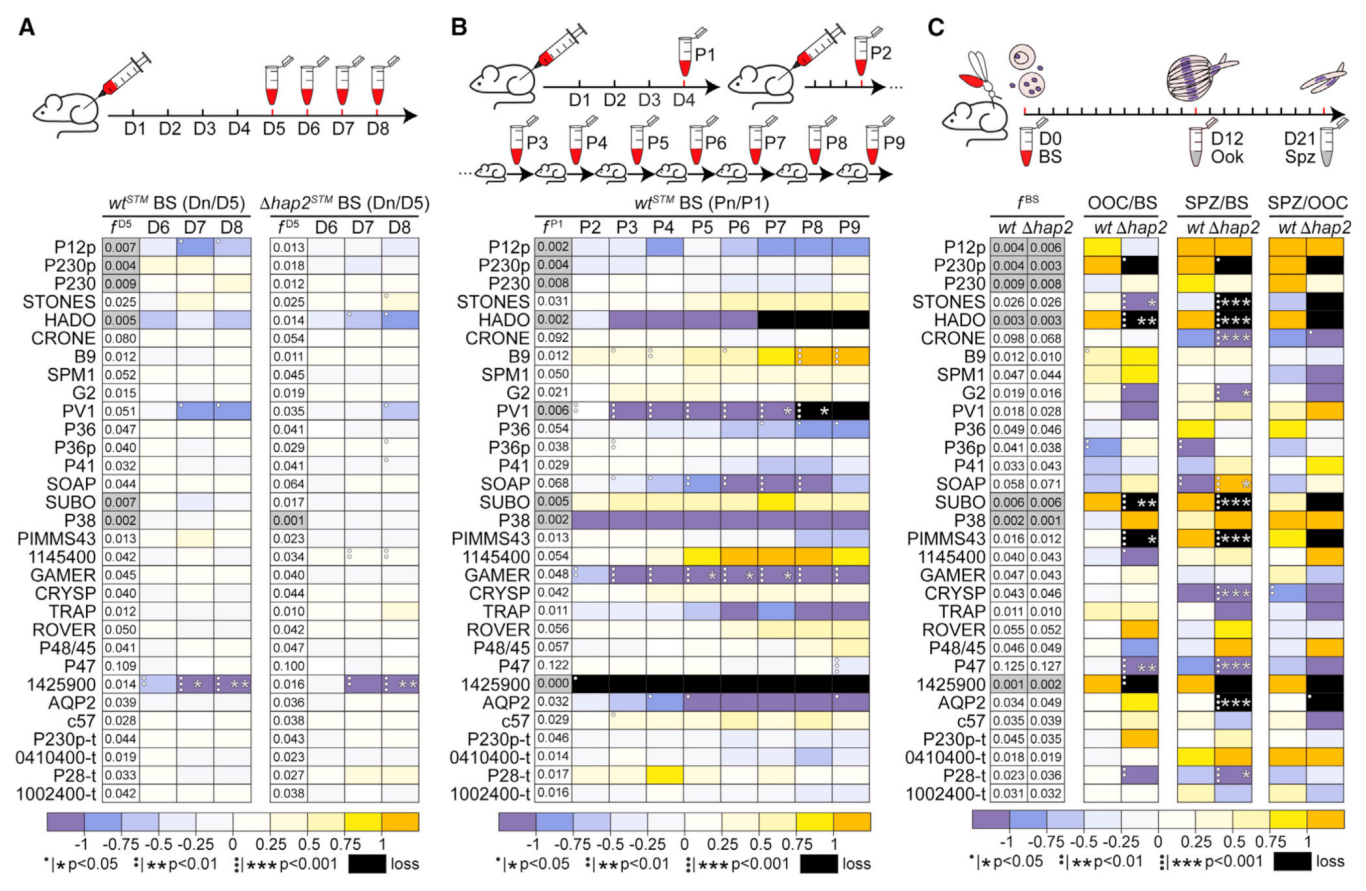
Results of STM screen (A) Fitness of blood stage STM mutants in WT (left) and *Δhap2* (right) genetic backgrounds shown as relative barcode abundance (ratio of each to total barcode counts) at days 5–8 (D5–D8) to day 5 (D5) post mouse infection. *f* ^day 5^ (*f* ^D5^) is frequency of each barcode in every 1,000 barcodes in day 5 (D5). (B) Stability of STM mutants in WT genetic background throughout 9 mouse-to-mouse passages (P1–P9) shown as relative barcode abundance in each passage (Pn) to the first passage (P1). *f* ^P1^ is frequency of each barcode in every 1,000 barcodes in P1. (C) Developmental progression of STM mutants in WT or *Δhap2* genetic backgrounds in *A. coluzzii* mosquitoes shown as relative barcode abundance in oocysts or sporozoites to blood stages, and sporozoites to oocysts. *f* ^BS^ is frequency of each barcode in every 1,000 barcodes in blood stages. Abundance differences in heatmaps are color-coded as in key; gray-shaded boxes represent barcodes with starting frequencies < 0.01; black boxes indicate zero or near zero count ratios. Statistical analysis done with Student’s t test, with p values shown as dots prior to multiple testing correction and as stars post multiple testing correction. ABSs, asexual blood stages; BSs, blood stages; Gam, gametocytes or gametes; Ook, ookinetes; Ooc, oocysts; Spz, sporozoites; WT, c507.

We assessed the fitness of each STM mutant parasite in the mouse host, expressed as relative barcode abundance (ratio of counts of each barcode to all barcodes) in days 5–8 compared with day 5 pi (Figure 3A; Table S2). This was done both in the validated Δ*hap2* and also in a WT genetic backgrounds, which would allow us to identify and exclude any genetic interactions between the pooled genes and *HAP2*.^25^ As expected, parasites lacking the gene PBANKA_1425900 that was previously reported to have a reduced ABS growth rate,^11^ which was used here as a control, exhibited drastically reduced fitness in both the WT and *Δhap2* genetic backgrounds, which remained statistically significant after false discovery rate (FDR) correction at days 7 and 8 pi. The abundance of all other mutants did not change significantly throughout the course of the infection.

Because *P. berghei* lines must be serially maintained in rodents either through direct blood passages until a fully replicated experiment is finished or with intermediate freezing and thawing of the infected blood, and although this presents an additional stress for the parasite, we wanted to examine the stability of the mutant parasite population during this process. We investigated the stability of the mutant population in the WT genetic background over 9 successive mouse-to-mouse passages (P1–9), by comparing the relative barcode abundance in each passage with that in the first infected mouse (Figure 3B; Table S2). Three mutants were found to drop out at different points of this experiment due to either reduced ABS growth rate or very low abundance in the pool following transfection: *PBANKA_1425900* (P2), *HADO* (P7), and *PV1* (P8). *HADO* encodes a putative magnesium phosphatase important for ookinete development, possibly by regulating the actin dynamics,^1^ and *PV1* encodes a homolog of an essential protein of the *Plasmodium falciparum* (*P. falciparum*) parasitophorous vacuole,^26^ likely an accessory of the Plasmodium translocon of exported proteins (PTEX) complex.^27^ Another mutant that started with a relatively high abundance (0.048) but was significantly depleted from the parasite population from P5 onward was *GAMER* (Figure 3C; Table S2). While *GAMER* is shown to be important for male gamete release,^1^ it is also highly expressed in ABS where it may have a non-essential or redundant role that eventually caused the loss of the mutant from the population due to fitness cost. From these results, we concluded that despite serial mouse passages having little or no impact on screening genes expressed in gametocytes onward, the parasite population should be better kept at a low passage stage to avoid the loss of mutants with low starting frequency. The minimum starting frequency was empirically set at 0.01.

### Oocyst and salivary gland sporozoite development in the mosquito host

The ability of the *P. berghei Plasmo*GEM mutants to infect *A. coluzzii* and develop into oocysts, which would then produce sporozoites that migrate and infect the salivary glands, was examined at days 12 and 21 pbf, respectively. Mutagenesis in both the WT and *Δhap2* genetic backgrounds was investigated; the *Δhap2* female-donor mutants were being crossed to the male-donor *Δnek4* through mouse co-infections. The ratio of normalized barcode counts in oocysts and salivary gland sporozoites to mouse blood stages prior to mosquito blood feeding was calculated for every mutant in four independent replicates. Data analysis revealed a stark difference between the two genetic backgrounds: while several *Δhap2 Plasmo*GEM mutants appeared to be significantly affected or completely dropped out from the screen at either or both of these stages, no significant effect was observed for any mutant in the WT background, likely due to complementation with a functional allele upon fertilization (Figure 3C).

Three mutants (*Δhado*, *Δsubo*, and *Δpimms43*) completely dropped out and another two mutants (*Δsto* and *Δp47*) were drastically depleted from the parasite population at the oocyst stage (Figure 3C). *Δsto* that carries a disruption of a previously unstudied gene, *STONES*, completely dropped out from the population in the salivary gland sporozoite stage. As said above, *HADO* is required for ookinete development and transformation to oocyst, with a possible role in midgut invasion.^1^ Both *PIMMS43* and *P47*^28^ are essential for ookinete protection from mosquito complement reactions upon midgut traversal.^29,30^ P47, a 6-cys domain protein also found on the surface of female gametocytes, is additionally important for gamete fertilization.^28,30^ *SUBO* (aka *PIMMS2*) encodes an ookinete-specific subtilisin-like protein required for ookinete traversal of the midgut epithelium, possibly being involved in epithelial cell egress.^31^ Two additional mutants, *ΔPBANKA_1425900* and *Δp230p*, also dropped out from the screen at this stage. P230p is a paralog of P230, a 6-cys domain protein involved in fertilization, and its *P. falciparum* ortholog is shown to be important for early parasite development in the mosquito.^32^

Five mutants showed reduced salivary gland sporozoite but normal oocyst growth. *Δaqp2* that carries a disruption of *AQP2*, a gene encoding a protein with high similarity to aquagly-ceroporins, completely dropped out from the salivary gland sporozoite population, while the relative abundances of *Δcro* (disruption of *CRONE*), *Δcry* (disruption of *CRYSP*), *Δg2* (disruption of *G2*), and *p28-t* (tagged *P28*) were significantly reduced (Figure 3C). *CRONE* (PBANKA_0720900), which encodes a 265 amino acid protein with an N-terminal signal peptide, has been previously shown to be expressed in gametocytes, where it is translationally repressed by DOZI, and translated in ookinetes to a protein localized in the crystalloids and required for sporozoite production in the oocyst.^7^ *CRYSP* (PBANKA_1338100) encodes a previously unstudied 263 amino acid protein with 3 transmembrane domains. The ookinete and sporozoite protein G2 (glycine at position 2) has been previously shown to localize in the cortical subpellicular network of these zoite stages and to be essential for their morphogenesis.^33^ Finally, *p28-t* is designed to produce a C-terminally 3x human influenza hemagglutinin (3xHA)-tagged version of the ookinete P28 Glycosylphosphatidylinositol (GPI)-anchored protein, a known target of transmission-blocking vaccines,^34^ and was used here as a negative control. Although P28 is known to have a redundant function, it appears that its tagging leads to a malfunctional protein that affects parasite development in the vector. Indeed, a drastic reduction of *p28-t* was already seen in the oocyst stage, although this was not statistically significant following FDR correction.

### Detailed phenotypic analysis of single mutant parasites

We selected for further analysis, three genes with strong phenotypes, *STONES, CRYSP*, and *CRONE*, as well as two genes for which the screen revealed no phenotype, *ROVER* and *SPM1*; an independent preliminary study had indicated that *ROVER* may be involved in infection, while *SPM1* was used as a control. *In silico* analysis of each of the predicted proteins and amino acid sequence alignments of selected orthologs from other *Plasmodium* species are presented in Figures S2–S6, respectively. Briefly, in addition to *CRYSP* and *CRONE* described earlier, *STONES* encodes a 1,037 amino acid protein with 14 transmembrane domains and coupled N-terminal LIS1 homology (LisH) motifs, thought to contribute to the regulation of the microtubule dynamics; *ROVER* encodes a 367 amino acid protein with no predicted domains; and *SPM1* encodes a 329 amino acid putative subpellicular microtubule (SPM) protein predicted to contain a microtubule-associated protein 6 (MAP6) domain.

We used the *Plasmo*GEM disruption vectors for *STONES* and *CRYSP* (Figure S1B) and conventional disruption vectors for *CRONE, ROVER*, and *SPM1* (Figure S1C) to generate clonal *P. berghei* mutants in the *c507* GFP-expressing WT line. Integration of the disruption cassettes and gene deletion in the clonal *Δsto*, *Δcry*, *Δcro*, *Δrov* (*ROVER*), and *Δspm1* parasite lines were confirmed by PCR (Figure S1).

**Figure 4:**
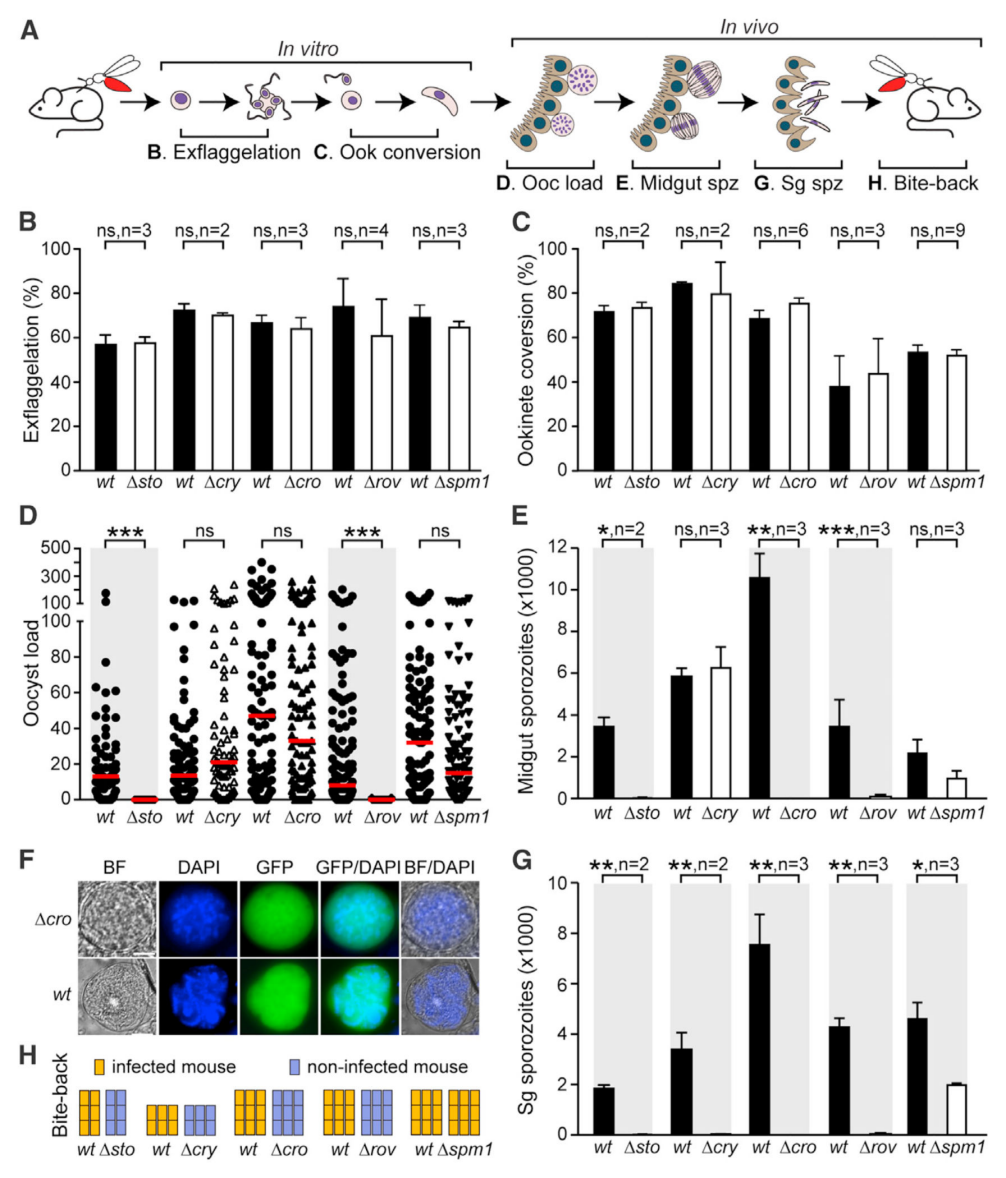
Phenotypic characterization of *P. berghei* knockout mutant parasites (A) Schematic representation of experimental assays. (B) Exflagellating male gametocyte percentage, compared with that of WT controls. Student’s t test used for statistical analysis. (C) Female gamete to ookinete conversion percentage, compared with WT controls. Statistical analysis done with Student’s t test. (D) Oocyst load in *A. coluzzii* midguts 8 days pbf, compared with WT controls. Red lines show median. Mann-Whitney is used for statistical analysis. (E) Sporozoite (Spz) numbers in *A. coluzzii* midguts of mutant parasites and WT controls. Student’s t test used for statistical analysis. (F) Fluorescence microscopy images of GFP-expressing *Δcro* oocysts compared with WT controls in *A. coluzzii* midguts 15 days pbf. DNA stained with DAPI. BF, bright field. Scale bars: 5 μM. (G) Sporozoite (Spz) numbers in *A. coluzzii* salivary glands (Sg) of mutant parasites, compared with WT controls. Student’s t test used for statistical analysis. (H) Mouse infection from bite-back of mosquitoes infected with mutant parasites or WT controls. Each mouse shown as a rectangle, columns indicate independent replicates. Infected mice shown in yellow, and non-infected mice shown in blue. In all panels: WT, *c507* line; ns, not significant; n, number of biological replicates; whiskers show SEM; *p < 0.05, **p < 0.001, ***p < 0.0001.

For all mutant parasites, we conducted a series of assays to investigate their phenotypes throughout the entire parasite life cycle in the mosquito vector (Figure 4A). Male gametogenesis, determined as the number of *in vitro* recorded exflagellation events per the number of male gametocytes, was comparable to that of the parental *c507* WT control (Figure 4B). Ookinete conversion rates, i.e., the ratio of *in vitro* produced ookinetes to female gametes counts, for all mutants were also not significantly different from that of the control(Figure 4C). Next, we examined the ability of the mutant parasites to form oocysts in *A. coluzzii*, following feeding of mosquitoes on mice infected with each of these mutants or the *c507* WT control line. Oocyst counts were determined 8 days pbf. *Δcry*, *Δcro*, and *Δspm1* mutants produced oocysts that were not significantly different in number from the control (Figure 4D; Table S3). However, a 99% decrease of mean oocyst numbers was observed for *Δsto* and *Δrov* mutants, with *Δrov* showing a maximum of only one oocyst in some midguts.

While the *STONES* phenotype was consistent with that obtained from the screen, the *ROVER* phenotype was unexpected and could only be justified by re-expression of the gene in the oo-kinetes and male WT allele rescue of the phenotype in the screen. We investigated this by crossing *Δrov* to either the female-donor *Δhap2* or the male-donor *Δnek4*, followed by oocyst counting in *A. coluzzii* 8 days pbf on co-infected mice. The *Δc57* line that harbors a disruption of *PIMMS57* was also included in these assays, as the screen also failed to detect this gene that has been previously shown to be important for ookinete-to-oocyst transition.^14^ The results confirmed that the oocyst-deficient phenotypes of both genes can be rescued by both the male and female WT alleles (Figure S7; Table S4), consistent with the expected limitation of the screen to reveal recessive phenotypes in diploid cells, after the WT allele introduced into the zygote by the microgamete is transcribed. *PIMMS57* is known to be specifically expressed in ookinetes,^14^ likely by both parental alleles, and the results suggest that the gametocyte-enriched *ROVER* gene is also expressed in ookinetes and that this expression is important for its function.

Next, we assessed the capacity of mutant parasites to produce sporozoites that can migrate to the salivary glands, by counting midgut (oocyst) and salivary gland sporozoites 15 and 21 days pbf, respectively. Consistent with a defective ookinete-to-oocyst transition, very few *Δsto* (10 ± 7) and *Δrov* (61 ± 11) oocyst sporozoites were detected (Figure 4E; Table S5), while the *Δcro* oocysts were also devoid of sporozoites, consistent with what has been reported previously.^7^ As the screen did not detect any significant reduction in oocyst *CRONE* barcode counts, we carried out microscopy on mature *Δcro* oocysts 15 days pbf to further investigate this phenotype. The results revealed that *Δcro* oocysts had large nuclei filled with DNA, but these were highly disorganized, unlike WT oocysts that exhibited highly organized nuclei with haploid sporozoites budding off from the sporoblastoid body (Figure 4F). These data indicated normal DNA replication in *Δcro* oocysts, but defective sporozoite formation and budding, further validating our genetic screen. Finally, the numbers of *Δcry* and *Δspm1* midgut sporozoites were not significantly different from those of WT *c507* controls, also corroborating the results obtained from the screen that showed that *cry* and *spm1* barcode counts in oocyst samples were not different from those detected in blood stages.

These results were also reflected in the salivary gland sporozoite counts for *Δsto*, *Δcro*, and *Δrov*, which ranged from very few to none (Figure 3G; Table S5). Importantly, and consistent with the results of the screen, none of the thousands of *Δcry* oocyst sporozoites were capable of infecting the salivary glands, again corroborating the results of the screen. A statistically significant 57% reduction in sporozoite counts was detected for *Δspm1*, suggesting that the effect seen in midgut sporozoites may also be true.

Finally, the ability of mutant parasites to transmit to the mouse host and infect red blood cells (RBCs) was assessed using mosquito-to-mouse (C57BL/6 strain) bite-back infections 21 days pbf (Figure 2H; Table S5). As expected, no transmission and development of mouse parasitemia was detected for any of the *Δsto*, *Δcro*, *Δcry*, and *Δrov* mutants, leading us to conclude that loss of function of the respective proteins leads to malaria transmission blockade. However, the reduction seen in *Δspm1* salivary gland sporozoites did not bear any impact on the capacity of mutant sporozoites to infect the mouse host, suggesting that SPM1 is dispensable for sporozoite development and transmission.

### STONES and ROVER are required for ookinete motility and mosquito midgut invasion

The endogenous *STONES, ROVER, CRYSP*, and *CRONE* genes were tagged with C-terminal 3xHA tag via double-crossover homologous recombination in the *c507* line, and the resulting transgenic lines were designated *stones::3xha, rover::3xha, crysp:: 3xha*, and *crone::3xha*, respectively (Figure S8).

**Figure 5:**
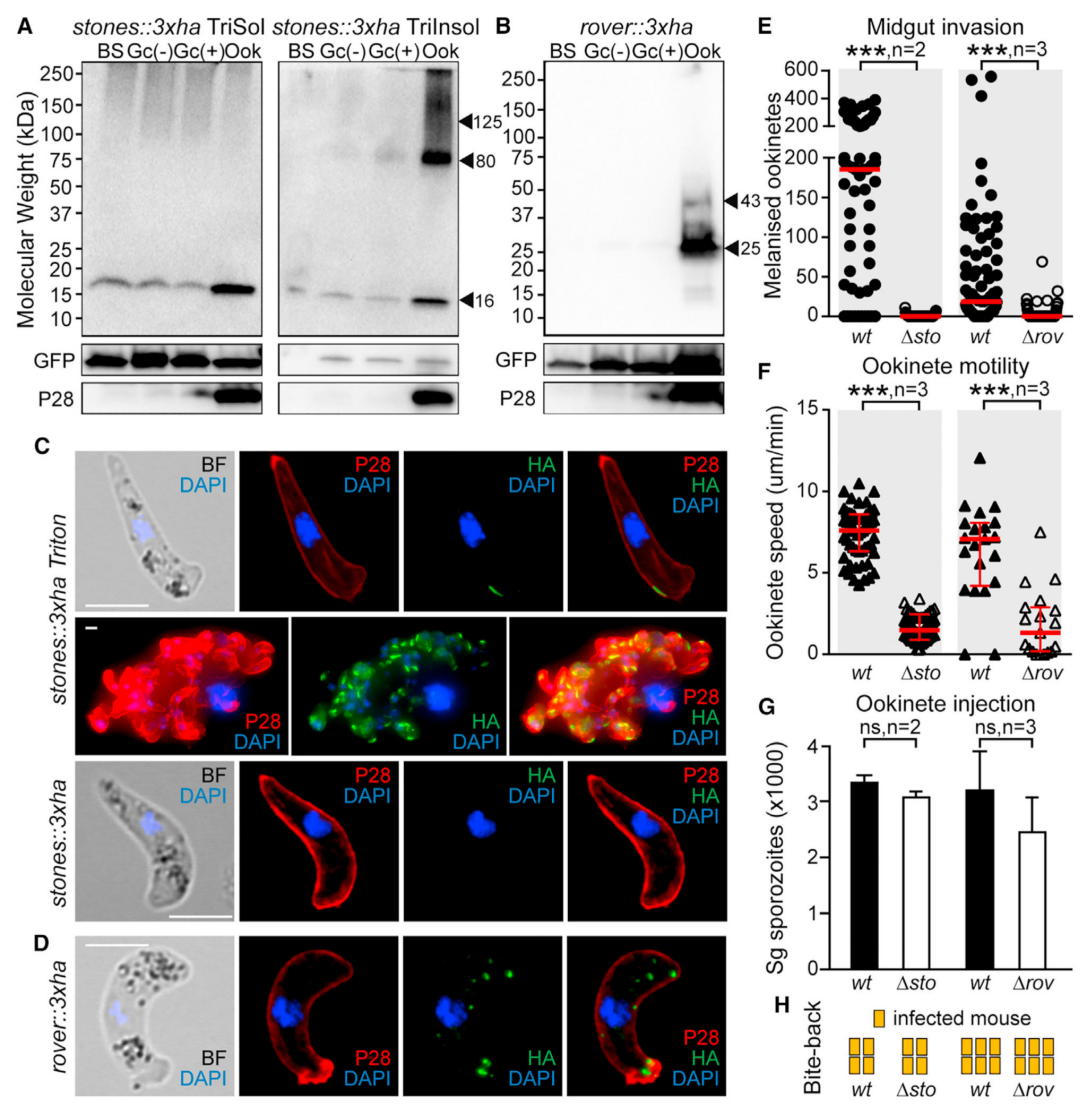
STONES and ROVER role in ookinete gliding motility (A) Western blot analysis in *stones::3xha* line using an α-HA antibody under reducing conditions on Triton X-100 soluble (TriSol) and Triton X-100 insoluble (TriInsol) fractions. STONES::3xHA-specific signals indicated with black arrowheads. GFP and P28 used as loading and stage-specific controls, respectively; BS, mixed blood stages; Gc(–), inactivated purified *in vitro* cultured gametocytes; Gc(+), activated purified *in vitro* cultured gametocytes; Ook, purified *in vitro* cultured ookinetes. (B) Western blot analysis in *rover::3xha* line using α-HA antibody under reducing conditions on whole-cell lysates. ROVER::3xHA-specific signals are indicated with black arrowheads. Abbreviations as above. (C) Immunofluorescence assays on *stones::3xha in vitro* cultured ookinetes Triton X-100 permeabilized (top two rows) and non-permeabilized (bottom row). Ookinetes stained with α-HA and α-P28 antibodies. DNA stained with DAPI. BF, bright field. Scale bars: 5 μM. (D) Immunofluorescence assays on *rover::3xha in vitro* cultured ookinetes stained with α-HA and α-P28 antibodies. DNA stained with DAPI. BF, bright field. Scale bars: 5 μM. (E) Numbers of melanized ookinetes in *CTL4* knockdown *A. coluzzii* infected with *Δsto*, *Δrov*, and *c507* WT controls. Red lines indicate median. Statistical analysis done with the Mann-Whitney test. ***p < 0.0001; n, number of biological replicates. (F) Speed of *in vitro* cultured *Δsto*, *Δrov*, and *c507* WT ookinetes measured with time-lapse microscopy (1 frame/5 s for 10 min). Horizontal red lines show median, and red whiskers show SEM. Statistical analysis done with the Mann-Whitney test; ***p < 0.0001; n, number of biological replicates. (G) Sporozoites numbers in *A. coluzzii* salivary glands (Sg) after hemocoel injection of *Δsto*, *Δrov*, and *c507* WT *in vitro* cultured ookinetes whiskers show SEM. Statistical analysis done with Student’s t test (unpaired two-tailed, equal variance); ns, not significant; n, number of biological replicates. (H) Bite-back mouse infection with mosquitoes infected with *Δsto*, *Δrov*, and *c507* WT controls. Each mouse shown as a rectangle, and columns indicate independent biological replicates. Infected mice shown in yellow.

We first analyzed *STONES* and *ROVER*, of which the disruption leads to defective ookinete phenotypes. The STONES:: 3xHA protein could not be detected at the predicted size of ~125 kDa in Triton X-100 soluble extracts of blood stages, gametocytes, or mature ookinetes. Instead, a band size of ~16 kDa was detected predominantly in mature ookinetes and less in blood stages and gametocytes (Figure 5A). However, in Triton X-100 insoluble extracts, a band of ~80 kDa was specifically detected in mature ookinetes, with traces of it also seen in gametocytes. As the full-length protein was never detected in any of the extracts, these results suggest that still ookinetes on the extrados site (STONES) undergoes proteolytic processing and that its C-terminal ~80-kDa fragment is embedded within the membrane owing to the multiple transmembrane domains. The ROVER::3xHA protein was detected only in mature ookinetes as 2 bands: the first at the expected size of ~43 kDa and the second, more predominant band at ~25 kDa (Figure 5B). This may indicate proteolytic cleavage of the protein.

In immunofluorescence assays, STONES::3xHA was specifically detected at a distinctive membrane region located on the convex side of the mature ookinete, posterior to the apical structure (Figure 5C). This region is critical for ookinete motility and has been termed OES.^35^ In non-Triton X-100 treated mature ookinetes, no signal at the OES could be detected, suggesting that the N-terminal HA-tagged part of STONES is intracellular, which is consistent with its topology predictions. ROVER::3xHA was localized in discrete cytoplasmic spots of mature ookinetes, resembling exocytic vesicles, commonly but not always positioned toward the apical end and in proximity to the cell membrane (Figure 5D).

The ookinete to oocyst defective phenotypes of the *Δsto* and *Δrov* parasites were further investigated in midgut invasion assays using a system we developed previously and that involved infections of *A. coluzzii* silenced for *CTL4*.^30,31^ CTL4 is a key hemolymph regulator of melanization, and its silencing leads to readily melanized *P. berghei* ookinetes that have succeeded in invading the midgut epithelium and reached the sub-epithelial space.^36^ The results showed that both *Δsto* and *Δrov* ookinetes displayed a great defect in midgut invasion as the number of melanized ookinetes were significantly reduced, compared with WT controls (Figure 5E; Table S6).

Defective midgut invasion can be due to the inability of ookinetes to move, and we assessed this by measuring the forward speed of ookinetes on matrigel. The results confirmed that both *Δsto* and *Δrov* mutants exhibit strong motility defects that likely cause their decreased ability to traverse the midgut epithelium and form oocysts and sporozoites (Figure 5F). To further examine this, *Δsto* and *Δrov* ookinetes were injected directly into the hemocoel to assess if the oocyst and sporozoite defective phenotypes could be rescued. Indeed, it has been previously shown that mosquito transmission of *P. berghei* mutants with ookinete motility defects can be rescued if midgut invasion is by-passed.^37^ The result confirmed that this was the case for both *Δsto* and *Δrov*, as both the salivary gland sporozoite numbers and the ability of mutants for mouse transmission through bite-back were restored (Figures 5G and 5H; Table S7).

Gliding motility is served by the glideosome, an actomyosin-based machinery located between the parasite plasma membrane (PPM) and the inner membrane complex (IMC).^38^ Initiation of gliding in mature ookinetes coincides with the polarization of the PPM protein guanylate cyclase β (GCβ) to the OES.^35^ This leads to local elevation of cyclic guanosine monophosphate (cGMP) levels and activation of cGMP-dependent protein kinase G (PKG) signaling that drives a series of events initiating gliding.^39^ Anchoring of GCβ and its co-factor CDC50A at the OES is facilitated by the IMC sub-compartment protein 1 (ISP1), which together with ISP3 interacts with β-tubulin on the SPM, serving as tethers to maintain the proper SPM structure.^35,40^ However, it remains unclear what pulls GCβ/CDC50A to the OES in the first place, as ISP1 polarizes already at the zygote stage. Also, ISP1 is required for GCβ/CDC50A polarization in the majority but not in all of the ookinetes, suggesting that additional proteins are involved in this process. The discovery of STONES, a multi-transmembrane protein of the OES, may help shed new light into the mechanisms enabling this critical step in malaria transmission. The presence of the LisH motifs suggests that STONES contributes to the regulation of the SPM dynamics, either by mediating dimerization or by binding SPM directly. While the STONES loss-of-function phenotype, cellular localization, and predicted SPM association appear to be closely matching those of ISP1, our data cannot clarify whether STONES, like ISP1, is integral to the IMC or the PPM.

Ookinetes lack rhoptries and dense granules, and most of the proteins important for gliding motility and midgut invasion are trafficked to the membrane or extracellularly through the micronemes. These are specialized secretory organelles that are synthesized *de novo* in the Golgi and translocate apically using filamentous connections with the SPM.^41,42^ Likewise, the ookinete IMC is thought to be formed *de novo* starting at the apical pole, most likely from Golgi-derived vesicles,^43^ and observations in *Toxoplasma gondii* suggest that IMC recycling also happens.^44^ The cellular localization of ROVER (roaming’s over) indicates an association with such vesicular structures. As the protein lacks a signal peptide and is never seen distributed across the membrane, it is suggestive that it acts as a cytoplasmic adaptor involved in vesicle trafficking.

### CRYSP and CRONE are crystalloid proteins essential for sporozoite development

**Figure 6:**
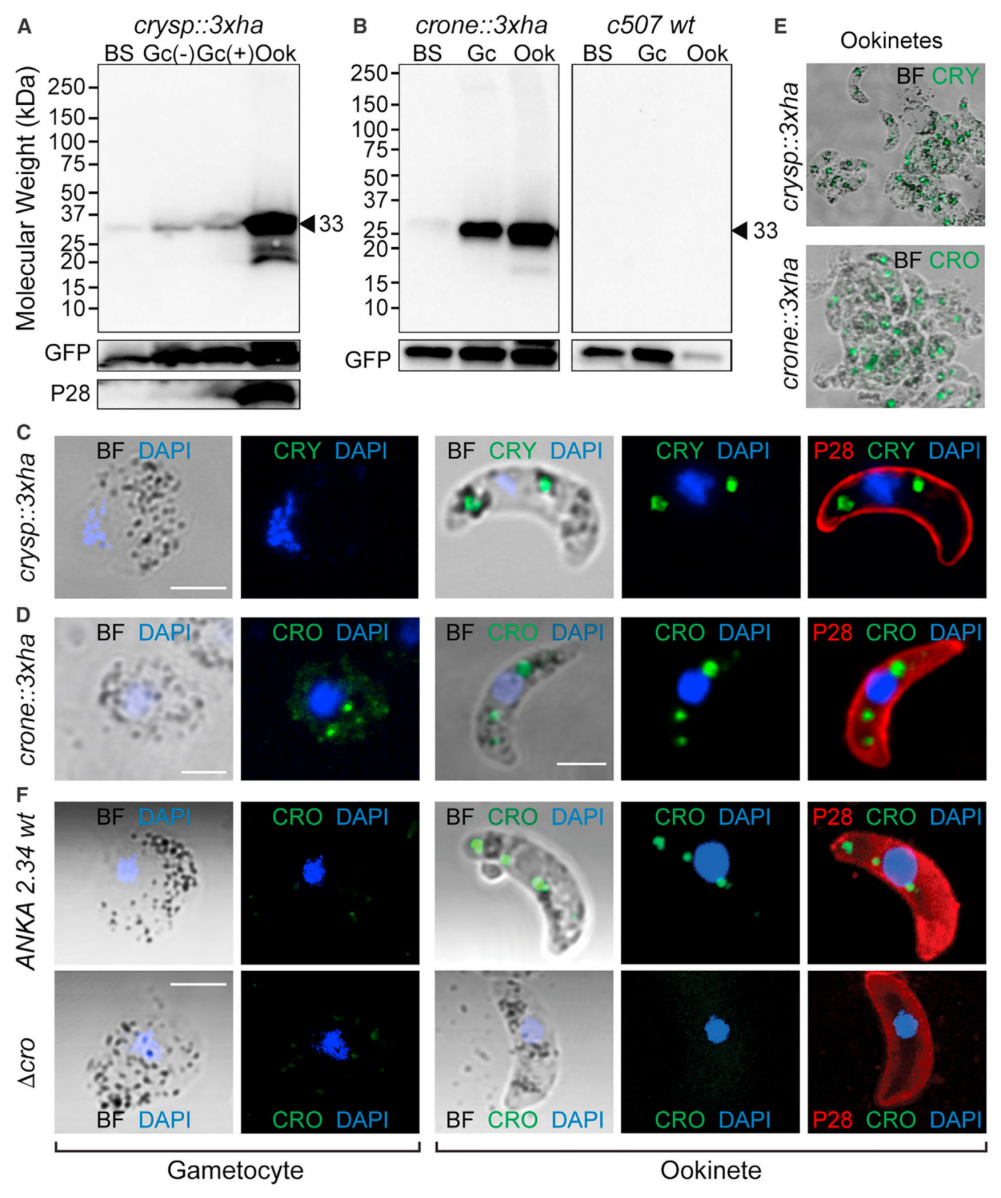
CRYSP and CRONE expression and localization (A) Western blot analysis of *crysp::3xha* and *c507* WT control using α-HA antibody under reducing conditions on whole-cell lysates. CRYSP::3xHA band is indicated with black arrowhead. GFP and P28 used as loading and stage-specific controls, respectively. BS, mixed blood stages; Gc(–), inactivated purified *in vitro* cultured gametocytes; Gc(+), activated and purified *in vitro* cultured gametocytes; Ook, purified *in vitro* cultured ookinetes. (B) Western blot analysis of *crone::3xha* (left) and *c507* WT control (right) using an α-HA antibody under reducing conditions on whole-cell lysates. CRONE::3xHA band is indicated with black arrowhead. Abbreviations as above. (C) Immunofluorescence assays of *crysp::3xha* purified gametocytes and ookinetes stained with α-HA and α-P28 antibodies. DNA stained with DAPI. BF, bright field. Scale bars: 2.5 μM. (D) Immunofluorescence assays of *crone::3xha* purified gametocytes and ookinetes stained with α-HA and α-P28 antibodies. DNA stained with DAPI. BF, bright field. Scale bars: 2.5 μM. (E) Immunofluorescence images of *crysp::3xha* and *crone::3xha* cultured ookinetes stained with α-HA. BF, bright field. (F) Immunofluorescence assays of *ANKA 2*.*34* WT and *Δcro* purified gametocytes and ookinetes stained with α-CRONE and α-P28 antibodies. DNA stained with DAPI. BF, bright field. Scale bars: 2.5 μM.

Western blot analyses using an anti-HA antibody detected the expected ~33 kDa CRYSP::3xHA protein in extracts from purified *in vitro* cultured *crysp::3xha* ookinetes and, at much lower levels, in gametocytes, both prior to and after induction of gametogenesis (Figure 6A). Similarly, the expected ~33-kDa CRONE::3xHA protein was detected in ookinete and, less so, in gametocyte extracts of the *crone::3xha* line (Figure 6B).

We examined the cellular localization of the two proteins in immunofluorescence assays of gametocytes and ookinetes (Figures 6C and 6D). In both cases, a clear and distinct spot pattern that always co-localized with the hemozoin (visible in bright field) was detected in the ookinete. This pattern is the hallmark of crystalloid localization in *P. berghei*.^45^ Multiple ookinete observations revealed that the number of spots varied from one to three, always in association with the hemozoin-containing vesicles, and were present in all the ookinetes observed (Figure 6E). The two proteins were henceforth named CRONE for “crystalloid oocyst not evolving” and CRYSP for “crystalloid needed for sporozoites.”

In the *crone::3xha* line, a vesicle-like, albeit less prominent, staining pattern was also detected in the female gametocytes, consistent with the high CRONE protein abundance in gametocyte extracts. Crystalloids are organelles known to be specific to ookinetes and young oocysts, thought to form soon after fertilization through active assembly of endoplasmic reticulum (ER)-derived vesicles.^10^ Some of the known crystalloid proteins are also synthesized in the gametocytes.^46^ Therefore, one can speculate that the CRONE::3xHA-stained gametocyte vesicles are crystalloid precursor subunits. While this may be true, the expression of CRONE in gametocytes could be due to the CRONE::3xHA expression design that used the *P. berghei* dihydrofolate reductase (DHFR) 3′ untranslated region (UTR). *Cis*-acting elements in the 5′ UTR or 3′ UTR of DOZI-regulated genes have been shown to be important for translational repression.^47^ Indeed, a previous study that expressed a GFP-tagged version of CRONE using the 3′ UTR of P28 that is also translationally repressed by DOZI found that GFP is restricted to the ookinete crystalloid.^7^ To examine this, we raised rabbit polyclonal antibodies against a codon-optimized fragment of CRONE (amino acids 24–235) expressed in *Escherichia coli* cells. Using these antibodies in immunofluorescence assays, we detected a clear ookinete crystalloid signal, but this signal was absent from gametocytes (Figure 6F). This indicated that the gametocyte signal detected in the *crone::3xha* line is likely due to leaky DOZI post-transcriptional repression.

Our findings add to the increasing recognition of the pivotal role of the crystalloid in sporogony and mosquito-to-human transmission. Although the details remain poorly understood, the current view is that the crystalloid assembles from ER-derived vesicles in a microtubule-dependent mechanism and serves in transporting functionally diverse proteins to the maturing oocyst.^10,46^ The commonly more than one and often two ookinete crystalloids appear as a single organelle in the oocyst,^46^ but it is unclear whether this is already a single multi-lobed organelle or is due to asynchronous dissolution or merging of separate crystalloids. The reason behind the transportation of proteins by this organelle instead of their contemporaneous synthesis in the oocyst is unclear; it may be attractive to speculate that during early stages of development, the metabolic environment in the oocyst is incompatible with *de novo* transcription and translation of proteins needed for sporogenesis. An alternative hypothesis is that this organelle functions in the ookinete and young oocyst with a knock-on effect in the mature oocyst.

The crystalloid founding molecules are the Limulus clotting factor C, Coch-5b2, and Lgl1 (LCCL)-lectin adhesive-like proteins (LAPs) that exhibit modular domain architectures implicated in protein, lipid, and/or carbohydrate binding.^10^ A recent proteomic analysis of *P. berghei* crystalloids revealed that the LAPs are part of an extended protein interaction network, which includes CRYSP and TPM2 (PBANKA_1104100), a structural homolog of CRONE.^48^ Both CRONE and TPM2 contain a TPM domain, named after its founding proteins, the *Arabidopsis thaliana* TLP18.3 and Psb32 and the *Caenorhabditis elegans* MOLO-1, as well as a C-terminal transmembrane domain. The TPM domain, despite being structurally conserved, exhibits varied functions. The absence of conserved amino acids required for the phosphatase activity of TLP18.3^49^ from CRONE and TPM2 may suggest different functions of these crystalloid proteins. It is yet unclear whether TPM2 shares the same pheno-type as CRONE, i.e., normal mitotic replication but failure of sporulation.

The discovery of CRYSP brings another perspective into the role of the crystalloid, as this is the first crystalloid protein unam-biguously shown to be involved in sporozoite egress from the oocyst or in infectivity rather than formation. A similar function has been previously suggested for the *P. falciparum* LAP orthologs, CCp2 and CCp3, but that study has not examined whether the seemingly normal oocyst sporozoites as seen with electron microscopy are fully formed and can be separated from the body of the oocyst.^50^ Indeed, this study did not detect sporozoites in the mosquito hemocoel. Disruption of LAP4, the *P. berghei* ortholog of PfCCp2, exhibits abnormal crystalloid biogenesis and gives rise to small and early sporulating oocysts that produce non-infectious sporozoites.^51^ In contrast, disruptions of LAP1, the *P. berghei* ortholog of PfCCp3, and of LAP3 produce parasites that are devoid of crystalloids and fail to complete oocyst maturation.^45,52^

In addition to the LAPs, CRONE, and CRYSP, the other two characterized crystalloid *P. berghei* proteins are the palmitoyl-S-acyl transferase DHHC10, thought to be involved in posttranslational lipid modification of proteins,^53^ and the nicotinamide adenine dinucleotide phosphate (NADP) transhydrogenase (NTH) that reduces NADP to NADPH.^48^ Both proteins are predicted to be transmembrane and are required for crystalloid biogenesis and sporozoite formation. Therefore, a common theme that emerges from these studies is that biogenesis of the crystalloids is dependent on the successful loading of most if not all their protein cargo. This would suggest that the sporogony-deficient phenotype of the mutants is an all-encompassing effect linked to the lack of the crystalloids rather than each individual protein. It remains to be seen whether this is true for CRONE and CRYSP, although the distinct phenotype of the latter suggests otherwise.

## Star★Methods

### Key Resources Table

**Table:** 

REAGENT or RESOURCE	SOURCE	IDENTIFIER
Antibodies		
Goat polyclonal anti-GFP	Rockland Immunochemicals	Cat# 600-101-215; RRID: AB_218182
Rabbit monoclonal anti-HA	Cell Signalling Technology	Cat# 3724S; RRID: AB_1549585
13.1 mouse monoclonal α-P28	This manuscript	N/A
Cy3 13.1 mouse monoclonal α-P28	This manuscript	N/A
HRP conjugated goat α-rabbit IgG	Promega	Cat# W4011; RRID: AB_430833
HRP conjugated goat α-mouse IgG	Promega	Cat# W4021; RRID: AB_430834
HRP conjugated donkey α-goat IgG	Promega	Cat# V8051; RRID: AB_430838
Alexa Fluor goat α-rabbit 488	ThermoFisher Scientific	Cat# A-11034; RRID: AB_2576217
Alexa Fluor goat α-mouse 568	ThermoFisher Scientific	Cat# A-11031; RRID: AB_144696
Rabbit polyclonal anti-CRONE	This manuscript	N/A
Bacterial and virus strains		
*E. coli* BL21 cells	New England Biolabs	Cat# C2530H
Biological samples		
*A. coluzzii* mosquitoes, Ngousso strain	BEI Resources	MRA-1301, MR4
Chemicals, peptides, and recombinant proteins		
CRONEopt-6XHIS	This manuscript	N/A
Critical commercial assays		
Amaxa P3 Primary Cell 4D-Nucleofector X Kit S	Lonza	V4XP-3032
4D-Nucleofector Core Unit	Lonza	AAF-1002B
4D-Nucleofector X Unit	Lonza	AAF-1002X
MiSeq Reagent Kit v2 (300-cycles)	Illumina	Cat# MS-102-2002
MiSeq Sequencing System	Illumina	N/A
*P. berghei* custom gene expression microarray	Agilent	GE 4X44K, G2514F, AMADID #020578
Agilent Low Input Quick Amp Labeling Kit	Agilent	Cat# 5190-2306
Agilent Gene Expression Hybridization Kit	Agilent	Cat# 5188-5242
Gene-Pix 4000B scanner	Molecular Devices	N/A
Matrigel assay kit	BD Biosiences	Cat# 356237
T7 high yield transcription kit	ThermoFisher Scientific	Cat# K0441
Deposited data		
Array data- *P. berghei* gametocyte-enriched transcripts *in vivo* in *A. coluzzii* infected midgut	This manuscript	Accession#: E-MTAB-12718 (https://www.ebi.ac.uk/biostudies/arrayexpress/studies/E-MTAB-12718)
Experimental models: Cell lines		
Arrayed library of *E. coli* TSA cells harboring linear plasmids containing *P. berghei *gene targeting vectors.	PlasmoGEM resource	https://plasmogem.umu.se/pbgem/home
Experimental models: Organisms/strains		
Mouse: BALB/c inbred (female)	Envigo	BALB/cOlaHsd
Mouse: C57BL/6 (female)	Charles River	C57BL/6JRj
Mouse: CD1 (female)	Charles River	CD1
*P. berghei:* wildtype cl15cy1 (ANKA 2.34)	BEI Resources Repository	cl15cy1
*P. berghei:* NGP (ANKA 2.33)	Sinden et al.^54^	N/A
*P. berghei:* wildtype cl15cy1 (c507)	Janse et al.^55^	507m6cl1 (RMgm-7 https://www.pberghei.eu)
*P. berghei:* PbmCherry	Burda et al.^56^	1804cl1 (RMgm928 https://www.pberghei.eu)
*P. berghei:* PbNEK4 knockout	RMgmDB	826cl1 (RMgm257 https://www.pberghei.eu)
*P. berghei:* PbMAP2 knockout	Howick et al.^15^	(RMgm-1203 https://www.pberghei.eu)
*P. berghei:* PbHAP2 knockout	This manuscript	(RMgm-5344 https://www.pberghei.eu)
Oligonucleotides		
Primers for barcode amplification and index tagging	This manuscript	N/A
Primers for generation of transgenic parasites and protein expression	This manuscript	N/A
Recombinant DNA		
Plasmid pL00018	N/A	MRA-787, MR4
Plasmid pL0035	Braks et al.^22^	MRA-850, MR4
CRONEopt	This manuscript	N/A
pET32b-CRONEopt	This manuscript	N/A
Software and algorithms		
Image J	https://imagej.nih.gov/ij	1.54d
GraphPad Prism	https://www.graphpad.com/	8.0
R	https://www.r-project.org	4.3.0
Gene-Pix Pro	Molecular Devices	6.1
GeneSpring GX	Agilent	12.6

### Resource Availability

#### Lead contact

Further information and requests for resources and reagents should be directed to and will be fulfilled by the lead contact George K. Christophides (g.christophides@imperial.ac.uk).

#### Materials availability

Parasite lines and other reagents produced by this study are available under a material transfer agreement for not-for-profit research and can be requested by the lead contact. Note that in laboratory stocks and reagents, the various genes studies here are often referenced with their temporary given codes, i.e., PBANKA_0413500 (STONES) is N350, PBANKA_1338100 (CRYSP) is N38, PBANKA_1353800 (ROVER) is c53, and PBANKA_0720900 (CRONE) is c72.

### Experimental Models and Subject Details

#### Mouse models

Three different *Mus musculus* mouse models used as referenced in the text and in the STAR Methods. These were inbred BALB/c (Envigo), C57BL/6 (Charles River) and CD1 (Charles River). All mice used were females and 7-8-week-old upon purchase. The laboratory mouse is a safe, versatile and convenient experimental model to study *P. berghei*, mosquito-malaria parasite interactions and malaria transmission. *In vitro* culturing alternatives for this parasite are not available, except for the ookinete stage. Specifically, this model was used for infection of mice with *P. berghei* stocks, mouse-to-mosquito transmission of *P. berghei* by mosquito blood feeding on parasitized mice, mosquito-to-mouse transmission of *P. berghei* by mosquito blood feeding on naive mice, and generation of P. berghei transgenic lines. Animals were purchased by external providers and maintained in our facilities in small individual ventilated cages (IVCs) of 5 mice for up to 2 or 3 weeks, including 1 week acclimatization: 2 weeks for mice infected intraperitonially and 3 weeks for mice infected by mosquito bites. All related procedures were reviewed and approved by the Imperial College Animal Welfare and Ethical Review Body (AWERB) and carried out in accordance with the Animal Scientifics Procedures Act (ASPA) 1986 Directive 2010/63/EU on the protection of animals used for scientific purposes, under a UK Home Office project license. Oversight of the work was provided by dedicated veterinarians.

#### Parasite models

*P. berghei* lines used were: the wildtype cl15cy1 line (ANKA 2.34); the constitutively GFP-expressing and selectable marker free 507m6cl1 (c507) line, which has the GFP under the control of the ef1a promoter and is integrated into the 230p (PBANKA_0306000) gene locus^55^; the non-gametocyte producer ANKA 2.33 line^54^; a selectable marker free HAP2 knockout line generated using the PbGEM-102303 vector and the parental reference line *1804cl1* (RMgm928) which expresses mCherry under the control of the HSP70 promoter^56^; and the Nek4 knockout line *826cl1* (RMgm257). The cultivation and purification of parasites were carried out as described.^54^

#### Mosquito models

The mosquitoes used were *A. coluzzii* (previously *A. gambiae* M form) of the N’gousso strain colonized from field-collected mosquitoes in 2006 in Yaoundé, Cameroon. Mosquitoes were reared and maintained at standard insectary conditions (27 ± 1 °C and 70 ± 5%) humidity on a 12:12 light/dark (L:D) cycle: 11.5 h full light of ~ 300 lux starting at 6 am and 11.5 h darkness starting at 6 pm, separated by 0.5 h dawn and dusk transitions, respectively.

### Method Details

#### DNA microarray hybridizations

The *P. berghei* Agilent oligonucleotide microarray platform has been described previously.^1^ Remapping of oligonucleotide probes on the latest *P. berghei* genome assembly and annotation release of PlasmoDB (version 35, released on 09/2022) showed that the microarray encompassed 4,288 of the 5,254 genes predicted in the *P. berghei* genome. Of these, 3,428 genes were the same as in the original array design, thus were represented by the same probes. The remaining 860 genes were products of merging or splitting of genes between various gene builds and were therefore represented by a different combination of probes compared with the original probe combinations. They were not considered further to prevent errors due to gene annotation issues.

For RNA isolation, 30–40 *A. coluzzii* midguts from 3 biological replicate infections with ANKA 2.34 and ANKA 2.33 were dissected at 1 h pbf in ice cold phosphate-buffered saline (PBS) and immediately immersed in TRIzol reagent (Invitrogen). Total RNA was extracted according to the manufacturer’s instructions and quantified using NanoDrop® ND-1000 Spectrophotometer (Thermo Scientific). 2 μg of total RNA were used for the generation and labelling of cRNA using the Agilent low RNA input fluorescence amplification kit according to manufacturer’s instructions. 2 μg of Cy3 (ANKA 2.33) and Cy5 (ANKA 2.34) labelled cRNA were mixed and hybridized on the microarrays using the Agilent *in situ* hybridization kit according to the manufacturer’s instructions. After washing, the hybridized microarrays were scanned using the Gene-Pix 4000B scanner (Axon Instruments). Grid alignment, registering spot signal intensity, estimation of local backgrounds and manual inspection of spot quality were carried out using Gene-Pix Pro 6.1. Data normalization was carried out using the locally weighted linear regression method (Lowess) in GeneSpring GX 12.6 (Agilent Technologies). Significant transcriptional differences stages were calculated using a one-way ANOVA with a *P*-value cut-off of 0.05, following correction with the Benjamini-Hochberg hypergeometric test.

#### Generation of *Plasmo*GEM pools

To determine the optimal pool size for detecting defective STM mutations in the mosquito vector, we developed a robust model of genetic drift that takes into account variable population sizes. Our model assumes that all barcodes start with the same frequency in the mouse host and that no barcodes are lost during blood stage development. By utilizing the binomial distribution, we calculated the expected frequencies of each barcoded mutant in the total population of mutants, considering the immune and other physiological responses of the mosquito. This model enabled us to track individual mutants through the ookinete-to-oocyst transition bottle-neck using the normal approximation, which is approximately 20 parasites for immune *A. coluzzii* (or 100 parasites for non-immune vectors). Based on this, we determined that pools containing around 30 mutants, with 4 replicates infecting approximately 50 mosquitoes each, would provide sufficient statistical power to detect differences of less than 5% in the proportions of mutant populations between any two conditions.

To generate the pools, *Plasmo*GEM vectors were combined in equal concentrations, and the mixture was digested with NotI to linearize the targeting vector. A total of 3.2 μg of the purified digested vector mix containing about 100 ng of each vector was used per transfection as previously described.^12^ Briefly, purified schizonts derived from *wt, map2ko* or *hap2ko* mCherry parasites was electroporated using the FI-115 program of the 4D nucleofector system (Lonza). Transfected schizonts were then injected intravenously into BALB/c mice, and drug selection of resistant parasites was carried out by the administration of pyrimethamine in the drinking water (70 μg/mL). Mouse infections following transfection was monitored daily by Giemsa staining of tail blood smears.

#### Library generation and barcode sequencing

Genomic DNA was extracted from mouse blood sampled at day 4-8 post infection with the transfected parasites and at day 4 post infection with serially passaged parasites, and from mosquito midguts and salivary glands at days 10-12 and 21 pbf, respectively, using phenol-chloroform extraction. *Plasmo*GEM vector specific barcodes were sequenced using Illumina MiSeq as described previously.^12^ Briefly, *Plasmo*GEM barcodes were amplified by PCR using the genomic DNA and the primers arg444 and arg445 (Table S8). The PCR amplicons were then used for a second PCR that introduced 5′ adaptors and multiplexing barcodes using primers shown in Table S8, and the resulting libraries were pooled at 100 ng per library and sequenced using the Illumina MiSeq Reagent Kit v2. After sequencing, barcode sequences were extracted from the output raw sequence file using a Perl script, counted, and their relative abundance (counts per 1000 barcodes) within each pool was determined. The time course fitness analysis involved 4 independent mouse infections and barcode sequencing assays, except for day 7, where only 3 datasets were analyzed due to a sequencing failure. For the time course stability analysis, we carried out 3 independent mouse infections and barcode sequencing for all time points. Additionally, we conducted 4 independent mosquito infection and barcode sequencing assays, except for the *wt* sporozoite samples, where only 3 datasets were included due to a sequencing failure. All raw data can be found in Table S2. Statistical analysis was performed using a student’s t-test followed by false discovery rate (multiple testing) correction.

The minimum frequency of barcodes in the pool that would prevent their susceptibility to random drift and potential loss from the host was determined empirically to be 0.01. Mutants with starting frequencies less than 0.01 tend to be lost over time from the mouse host, during serial mouse passages or result in inconsistent mosquito infections.

#### Mosquito transmission of transgenic parasite pools

At 8 days post transfection with the *Plasmo*GEM vector pool when mice parasitemia was 6-8%, blood was obtained from infected mice via heart puncture and mixed with blood derived from mice infected with the NEK4 knockout parasite with the same parasitemia in a ratio of 2:1. This mixture was used to infect BALB/c mice. At a parasitemia of 3-4%, mice were used in direct mosquito feeds. 30-50 mosquito midguts and salivary glands per biological replicate were dissected and collected for genomic DNA extraction.

#### Generation of single knockout transgenic parasites

For the generation of the *Δmap2* and Δ*hap2* background lines, we used the *Plasmo*GEM vectors PbGEM-111778 and PbGEM-102303, respectively. A total of 5 μg of each plasmid was used to transfect segmented *P. berghei* schizonts as previously described.^25^ Briefly, schizonts were electroporated using the FI-115 program of the Amaxa Nucleofector 4D, after which parasites were immediately injected intravenously into the tail vein of BALB/c mice. Transgenic parasites were selected with 0.07 mg/mL py-rimethamine (Sigma) in drinking water from day 1 pi. Disruption was confirmed in the resistant parasite populations by PCR and clonal lines were derived by limiting dilution. To allow the use of *Δmap2* and *Δhap2* as background lines in the screen, we induced excision of the resistance cassette from the genome using negative selection, through the administration of 5 fluorocytosine (1 mg/mL, Sigma) via the drinking water.^22^ This was possible because each resistance cassette carried a gene encoding the yFCU that counteracts the administered 5 fluorocytosine. Each mutant was finally re-genotyped to confirm correct excision of the resistance cassette and clonal lines were once again derived by limiting dilution.

For disruption of *STONES* and *CRYSP*, we used the PbGEM_230494 and PbGEM_058356 *Plasmo*GEM vectors, respectively. The targeting cassettes were released by NotI digestion resulting in 84% and 80% deletion of the CDS of *STONES* and *CRYSP* at the 5′ end. Partial (66%) knockout of *CRONE* and full knockout of *ROVER* and *SPM1* was carried out by double crossover homologous recombination in the c507 line. For this, EcoRI/BamHI 5′ homology arms and Apa/HindIII 5′ homology arms were amplified from genomic DNA using the primer pairs P1/P2 (588 bp), P5/P6 (728 bp) and P9/P10 (620 bp) and P3/P4 (573 bp), P7/P8 (558 bp) and P11/P12 (648 bp), respectively (Table S9). These fragments were cloned into the Pbs-TgDHFR vector with homology arms flanking a modified *Toxoplasma gondii* dihydrofolate gene (*TgDHFR/TS*) cassette that confers resistance to pyrimethamine. The gene targeting cassettes were released by ApaI/BamHI digestion. Transfection, drug selection of transgenic parasites and clonal selection by dilution cloning was carried out as previously described.^55^

#### Generation of tagged transgenic parasites

For the C-terminal 3xHA tagging of *STONES, CRYSP* and *CRONE* in the *c507* line, we used the *Plasmo*GEM vectors PbGEM012712, PbGEM058364 and PbGEM089977, respectively.^12^ The C-terminal 3xHA tagging of *ROVER* in the c507 line was generated by Gibson assembly. Firstly, a 694 bp 5′ homology arm ApaI fragment corresponding to the most 3′ region of the CDS and the 3XHA sequence was amplified using the primer pairs P30/P31 (Table S9). The 460bp *DHFR* 3′*UTR* SacII fragment was amplified from the pL00018 vector (MRA-787, MR4) using the primers P32/P33. An overlap PCR using both fragments was set up to generate the Apa/SacII *ROVER::3XHA::DHFR* 3′*UTR*. A 560 bp Xho/XmaI 3′ homology arm region corresponding to the 3′*UTR* of the gene was amplified using the primer pairs P34/P35 (Table S9). The *ROVER* fragments were cloned flanking the *hDHFR/yFCU* selection cassette into plasmid pL0035.^22^

#### Genotypic analysis of transgenic parasites

Following drug selection and clonal selection, parasite genomic DNA was extracted from blood sampled from parasite positive mice using the DNeasy kit (Qiagen). Successful gene modification events or maintenance of the wildtype locus was performed by PCR using primers listed in Table S9.

#### Genetic crosses

Genetic crosses between the *Δrov* or *Δc57* and the *Δhap2* (male-deficient) or *Δnek4* (female-deficient) lines were carried out by infecting mice with different combinations of mutant parasites, which were then used for direct feeding of *A. coluzzii* mosquitoes.

#### Exflagellation assays

Blood from infected mice exhibiting 8-10% gametocytemia was added to RPMI medium (RPMI 1640, 20% v/v FBS, 100 μM xanthurenic acid, pH 7.4) in a 1:40 ratio and incubated for 10 min. Male exflagellation events were counted in a standard hemocytometer under a light microscope and compared to male gametocytemia as determined by microscopic observations of Giemsa blood smears.

#### Macrogamete to ookinete conversion assays

Ookinete formation was assessed by conversion assays. Blood from infected mice exhibiting 8-10% gametocytemia was added to RPMI medium (RPMI 1640, 20% v/v FBS, 100 μM xanthurenic acid, pH 7.4) and incubated for 24 hours at 21°C to allow for ookinete formation. This suspension was then incubated with a Cy3-labelled 13.1 mouse monoclonal α-P28 (1:50 dilution) for 20 min on ice.

The conversion rate was calculated as the percentage of Cy3 positive ookinetes to Cy3 positive macrogametes and ookinetes.

#### Ookinete motility assays

A 24-hour *in vitro* culture of mature ookinetes was added to Matrigel (BD biosciences), and the mixture was dropped onto a slide and allowed to set at room temperature for 30 min. Time-lapse microscopy (1 frame every 5 seconds, for 10 min) of ookinetes were taken on a Leica DMR fluorescence microscope and a Zeiss Axiocam HRc camera controlled by the Axiovision (Zeiss) software. The speed of individual ookinetes was measured using the manual tracking plugin in the Icy software package.

#### Invasion assay

Total RNA was extracted from *A. coluzzii* midguts infected with *P. berghei* 24 hours pbf using the TRIzol reagent (Invitrogen). The RNA was used to generate cDNA that was subsequently used in the amplification of *CTL4* using primers P51/P52 with T7 overhangs to produce double-stranded RNA using the T7 high yield transcription kit (ThermoFisher). The double-stranded RNA was purified using the RNeasy kit (Qiagen) and 0.2 μg in 69 nL was injected through the mesothoracic spiracle into the hemocoel cavity of *A. coluzzii* mosquitoes using glass capillary needles and the Nanoject II microinjector (Drummond Scientific). Injected mosquitoes were allowed to recover for 3 days before blood feeding.

#### *P. berghei* mosquito infections

Mosquitoes were infected by direct feeding on mice infected with *P. berghei* at a parasitemia and gametocytemia of 5-6% and 1-2%, respectively. To determine oocyst load, midguts were dissected at 7-10 days pbf and fixed in 4% paraformaldehyde. Melanized parasites and oocyst numbers were counted under a light and fluorescent microscope, respectively. To determine sporozoite load, 25-30 midguts and salivary glands were dissected 15 and 21 days pbf, respectively, homogenized and sporozoites counted in a standard hemocytometer under a light microscope. To assess mosquito-to-mouse transmission, about 30 *A. coluzzii* mosquitoes that had blood-fed on *P. berghei*-infected mice 20-22 days earlier were allowed to feed on 2-3 anaesthetized C57/BL6 mice. Mouse parasitemia was monitored until 14 days post mosquito bite by Giemsa staining.

#### Ookinete injection

The concentration of ookinetes obtained from a 24-hour *in vitro* ookinete culture was adjusted to achieve injection of approximately 800 ookinetes per mosquito delivered through injection of *A. coluzzii* females through the mesothoracic spiracle using glass capillary needles and the Nanoject II microinjector (Drummond Scientific). Salivary glands were dissected 21 days post injection and homogenized, and sporozoite numbers were counted using a standard hemocytometer under a light microscope.

#### Protein expression, purification, and antibody production

A *CRONE* CDS fragment corresponding to amino acids 23-235, which excludes the predicted N-terminal signal peptide and C-terminal transmembrane domain, was codon optimized for expression in *E. coli* (GeneArt, ThermoFisher). This fragment was amplified with the primer pair P49/P50 (Table S9) and cloned into a NotI digested pET-32b protein expression vector, which carries the N and C-terminal 6xHistidine tags (Novagen), using the Hi-Fi DNA assembly kit (New England Biosciences).

*E. coli* BL21 cells (New England Biosciences) containing the recombinant protein expression plasmid were grown at 37°C and induced with 1 mM isopropyl-1-thio-β-d-galactopyranoside at 18°C for 16 hours. Cells were harvested and lysed using cell lytic (Sigma) containing the protease inhibitors cOmplete EDTA-free (Roche). Cell debris were removed by centrifugation. The His-tagged CRONE protein was purified by cobalt affinity chromatography using TALON® metal affinity resin (Takara) under native conditions in phosphate buffered saline (PBS), pH 7.4. Protein samples were analyzed by SDS-PAGE to determine purity prior to their use for immunization in rabbits for the generation of an affinity purified polyclonal antibody (Eurogentec).

#### Western blot analysis

Western blot analysis was performed on whole cell lysates and fractionated cell samples. To extract whole cell lysates, purified blood stages, gametocytes and ookinetes were suspended in whole cell lysis buffer (1XPBS, 1% v/v Triton X-100). For fractionation, firstly, the soluble fraction was obtained by suspension and homogenization of purified parasites in soluble cell lysis buffer (50mM Tris, 300mM NaCl). Secondly, to obtain the Triton-Soluble fraction, the pellet from the prior treatment was then resuspended and homogenized in Triton-solubilization buffer (50mM Tris, 300mM NaCl, 1% v/v Triton X-100). Finally, to obtain the Triton-Insoluble fraction, the pellet from the prior treatment was resuspended and homogenized in Laemilli SDS buffer. Protein fractions were boiled under reducing conditions and separated using 4-20% sodium dodecyl sulfate (SDS) polyacrylamide gel electrophoresis. The gel separated proteins were transferred to a polyvinylidene difluoride (PVDF) membrane. Proteins were detected using rabbit α-HA (Cell Signaling Technology) (1:1000), goat α-GFP (Rockland chemicals) (1:1000) and 13.1 mouse monoclonal α-P28 (1:1000) antibodies. Secondary horseradish peroxidase (HRP) conjugated goat α-rabbit IgG, goat α-mouse IgG antibodies (Promega) and donkey α-goat IgG (Abcam) were used at 1: 2,500, 1: 2,500 and 1: 5,000 dilutions, respectively. All primary and secondary antibodies were diluted in 5% w/v milk-PBS-Tween (0.05% v/v) blocking buffer.

#### Indirect immunofluorescence assays

Blood stage gametocytes, ookinetes and sporozoites were fixed in 4% paraformaldehyde (PFA) in PBS for 10 min at room temperature. Fixed parasites were washed 3X with 1XPBS for 10 min each and then smeared on glass slides. Permeabilization of the parasites was done using 0.2% v/v Triton X-100 in PBS for 10 min at room temperature. Permeabilized parasites were washed 3 times in PBS for 10 min each and then blocked with 1% w/v bovine serum albumin in PBS for 1 hour at room temperature. Parasites were stained with rabbit α-HA (CST) (1:1000) and 13.1 mouse monoclonal α-P28 (1:1000) antibodies. Alexa Fluor rabbit 488 and mouse 568 conjugated secondary goat antibodies (ThermoFisher) were used at a dilution of 1:1000. 4′,6-diamidino-2-phenylindole (DAPI) was used to stain nuclear DNA. Images were acquired using a Leica SP5 MP confocal laser-scanning microscope. Images were visualized using Image J.

#### Statistical analyses

Statistical analyses were performed using GraphPad Prism v8.0 and Microsoft Excel. P-values for exflagellation, ookinete conversion and motility assays were calculated using a two-tailed, unpaired student’s t-test. Statistical analyses of the oocyst or melanized parasite infection intensities and presence of oocysts (infection prevalence), *P*-values were calculated using the Mann-Whitney test. Statistical analyses of the barcode sequencing data were performed using student’s t-test, followed by false discovery rate (multiple testing) corrections.

## Supplementary Material

Supplementary Material Supplemental information can be found online at https://doi.org/10.1016/j.chom.2023.08.010.

## Data Availability

The raw array data for this study have been deposited under accession number E-MTAB-12718 (https://www.ebi.ac.uk/biostudies/arrayexpress/studies/E-MTAB-12718). Accession numbers are also listed in the key resources table. Microscopy data reported in this paper will be shared by the lead contact upon request. Any additional information required to reanalyze the data reported in this paper is available from the lead contact upon request.

## In brief

Malaria remains a devastating disease, and new concepts are needed to block transmission between people. Ukegbu et al. present a genetic approach that uncovers genes and processes required for the malaria parasite’s developmental transition through the mosquito vector, such as motility and production of sporozoites that infect humans.

## Highlights

- Reverse genetic screen reveals *Plasmodium* genes required during mosquito infection
- Ookinete STONES and ROVER are required for motility and mosquito midgut invasion
- Crystalloid CRONE and CRYSP are essential for sporogony and sporozoite development
- Ookinete motility and crystalloids are candidate targets to block malaria transmission

## References

[1] Akinosoglou KA, Bushell ES, Ukegbu CV, Schlegelmilch T, Cho JS, Redmond S, Sala K, Christophides GK, Vlachou D (2015). Characterization of Plasmodium developmental transcriptomes in Anopheles gambiae midgut reveals novel regulators of malaria transmission. Cell Microbiol.

[2] Ukegbu CV, Cho JS, Christophides GK, Vlachou D (2015). Transcriptional silencing and activation of paternal DNA during Plasmodium berghei zygotic development and transformation to oocyst. Cell Microbiol.

[3] Modrzynska K, Pfander C, Chappell L, Yu L, Suarez C, Dundas K, Gomes AR, Goulding D, Rayner JC, Choudhary J (2017). A knockout screen of ApiAP2 genes reveals networks of interacting transcriptional regulators controlling the plasmodium life cycle. Cell Host Microbe.

[4] Yuda M, Iwanaga S, Shigenobu S, Mair GR, Janse CJ, Waters AP, Kato T, Kaneko I (2009). Identification of a transcription factor in the mosquito-invasive stage of malaria parasites. Mol Microbiol.

[5] Hall N, Karras M, Raine JD, Carlton JM, Kooij TW, Berriman M, Florens L, Janssen CS, Pain A, Christophides GK (2005). A comprehensive survey of the Plasmodium life cycle by genomic, transcriptomic, and proteomic analyses. Science.

[6] Lasonder E, Rijpma SR, van Schaijk BC, Hoeijmakers WA, Kensche PR, Gresnigt MS, Italiaander A, Vos MW, Woestenenk R, Bousema T (2016). Integrated transcriptomic and proteomic analyses of P. falciparum gametocytes: molecular insight into sex-specific processes and translational repression. Nucleic Acids Res.

[7] Guerreiro A, Deligianni E, Santos JM, Silva PA, Louis C, Pain A, Janse CJ, Franke-Fayard B, Carret CK, Siden-Kiamos I (2014). Genome-wide RIP-Chip analysis of translational repressor-bound mRNAs in the Plasmodium gametocyte. Genome Biol.

[8] Mair GR, Braks JA, Garver LS, Wiegant JC, Hall N, Dirks RW, Khan SM, Dimopoulos G, Janse CJ, Waters AP (2006). Regulation of sexual development of Plasmodium by translational repression. Science.

[9] Mair GR, Lasonder E, Garver LS, Franke-Fayard BM, Carret CK, Wiegant JC, Dirks RW, Dimopoulos G, Janse CJ, Waters AP (2010). Universal features of post-transcriptional gene regulation are critical for Plasmodium zygote development. PLoS Pathog.

[10] Dessens JT, Tremp AZ, Saeed S (2021). Crystalloids: fascinating parasite organelles essential for malaria transmission. Trends Parasitol.

[11] Bushell E, Gomes AR, Sanderson T, Anar B, Girling G, Herd C, Metcalf T, Modrzynska K, Schwach F, Martin RE (2017). Functional profiling of a plasmodium genome reveals an abundance of essential Genes. Genes Cells.

[12] Gomes AR, Bushell E, Schwach F, Girling G, Anar B, Quail MA, Herd C, Pfander C, Modrzynska K, Rayner JC (2015). A genome-scale vector resource enables high-throughput reverse genetic screening in a malaria parasite. Cell Host Microbe.

[13] Stanway RR, Bushell E, Chiappino-Pepe A, Roques M, Sanderson T, Franke-Fayard B, Caldelari R, Golomingi M, Nyonda M, Pandey V (2019). Genome-scale identification of essential metabolic processes for targeting the plasmodium liver stage. Cell.

[14] Ukegbu CV, Christophides GK, Vlachou D (2021). Identification of three novel plasmodium factors involved in ookinete to oocyst developmental transition. Front Cell Infect Microbiol.

[15] Howick VM, Russell AJC, Andrews T, Heaton H, Reid AJ, Natarajan K, Butungi H, Metcalf T, Verzier LH, Rayner JC (2019). The Malaria Cell Atlas: single parasite transcriptomes across the complete Plasmodium life cycle. Science.

[16] Billker O, Shaw MK, Margos G, Sinden RE (1997). The roles of temperature, pH and mosquito factors as triggers of male and female gametogenesis of Plasmodium berghei in vitro. Parasitology.

[17] Tewari R, Dorin D, Moon R, Doerig C, Billker O (2005). An atypical mitogen-activated protein kinase controls cytokinesis and flagellar motility during male gamete formation in a malaria parasite. Mol Microbiol.

[18] Tewari R, Straschil U, Bateman A, Böhme U, Cherevach I, Gong P, Pain A, Billker O (2010). The systematic functional analysis of Plasmodium protein kinases identifies essential regulators of mosquito transmission. Cell Host Microbe.

[19] Hirai M, Arai M, Mori T, Miyagishima SY, Kawai S, Kita K, Kuroiwa T, Terenius O, Matsuoka H (2008). Male fertility of malaria parasites is determined by GCS1, a plant-type reproduction factor. Curr Biol.

[20] Liu Y, Tewari R, Ning J, Blagborough AM, Garbom S, Pei J, Grishin NV, Steele RE, Sinden RE, Snell WJ (2008). The conserved plant sterility gene HAP2 functions after attachment of fusogenic membranes in Chlamydomonas and Plasmodium gametes. Genes Dev.

[21] Mori T, Hirai M, Kuroiwa T, Miyagishima SY (2010). The functional domain of GCS1-based gamete fusion resides in the amino terminus in plant and parasite species. PLoS One.

[22] Braks JA, Franke-Fayard B, Kroeze H, Janse CJ, Waters AP (2006). Development and application of a positive-negative selectable marker system for use in reverse genetics in Plasmodium. Nucleic Acids Res.

[23] Reininger L, Billker O, Tewari R, Mukhopadhyay A, Fennell C, Dorin-Semblat D, Doerig C, Goldring D, Harmse L, Ranford-Cartwright L (2005). A NIMA-related protein kinase is essential for completion of the sexual cycle of malaria parasites. J Biol Chem.

[24] Reininger L, Tewari R, Fennell C, Holland Z, Goldring D, Ranford-Cartwright L, Billker O, Doerig C (2009). An essential role for the Plasmodium Nek-2 Nima-related protein kinase in the sexual development of malaria parasites. J Biol Chem.

[25] Fang H, Gomes AR, Klages N, Pino P, Maco B, Walker EM, Zenonos ZA, Angrisano F, Baum J, Doerig C (2018). Epistasis studies reveal redundancy among calcium-dependent protein kinases in motility and invasion of malaria parasites. Nat Commun.

[26] Chu T, Lingelbach K, Przyborski JM (2011). Genetic evidence strongly support an essential role for PfPV1 in intra-erythrocytic growth of P. falciparum. PLoS One.

[27] Morita M, Nagaoka H, Ntege EH, Kanoi BN, Ito D, Nakata T, Lee JW, Tokunaga K, Iimura T, Torii M (2018). PV1, a novel Plasmodium falciparum merozoite dense granule protein, interacts with exported protein in infected erythrocytes. Sci Rep.

[28] van Dijk MR, van Schaijk BC, Khan SM, van Dooren MW, Ramesar J, Kaczanowski S, van Gemert GJ, Kroeze H, Stunnenberg HG, Eling WM (2010). Three members of the 6-cys protein family of Plasmodium play a role in gamete fertility. PLoS Pathog.

[29] Ukegbu CV, Giorgalli M, Tapanelli S, Rona LDP, Jaye A, Wyer C, Angrisano F, Blagborough AM, Christophides GK, Vlachou D (2020). PIMMS43 is required for malaria parasite immune evasion and sporogonic development in the mosquito vector. Proc Natl Acad Sci USA.

[30] Ukegbu CV, Giorgalli M, Yassine H, Ramirez JL, Taxiarchi C, Barillas-Mury C, Christophides GK, Vlachou D (2017). Plasmodium berghei P47 is essential for ookinete protection from the Anopheles gambiae complement-like response. Sci Rep.

[31] Ukegbu CV, Akinosoglou KA, Christophides GK, Vlachou D (2017). Plasmodium berghei PIMMS2 promotes ookinete invasion of the Anopheles gambiae mosquito midgut. Infect Immun.

[32] Marin-Mogollon C, van de Vegte-Bolmer M, van Gemert GJ, van Pul FJA, Ramesar J, Othman AS, Kroeze H, Miao J, Cui L, Williamson KC (2018). The Plasmodium falciparum male gametocyte protein P230p, a paralog of P230, is vital for ookinete formation and mosquito transmission. Sci Rep.

[33] Tremp AZ, Carter V, Saeed S, Dessens JT (2013). Morphogenesis of Plasmodium zoites is uncoupled from tensile strength. Mol Microbiol.

[34] Tomas AM, Margos G, Dimopoulos G, van Lin LH, de Koning-Ward TF, Sinha R, Lupetti P, Beetsma AL, Rodriguez MC, Karras M (2001). P25 and P28 proteins of the malaria ookinete surface have multiple and partially redundant functions. EMBO J.

[35] Gao H, Yang Z, Wang X, Qian P, Hong R, Chen X, Su XZ, Cui H, Yuan J (2018). ISP1-anchored polarization of GCβ/CDC50A complex initiates malaria ookinete gliding motility. Curr Biol.

[36] Osta MA, Christophides GK, Kafatos FC (2004). Effects of mosquito genes on Plasmodium development. Science.

[37] Guttery DS, Poulin B, Ferguson DJ, Szöör B, Wickstead B, Carroll PL, Ramakrishnan C, Brady D, Patzewitz EM, Straschil U (2012). A unique protein phosphatase with kelch-like domains (PPKL) in Plasmodium modulates ookinete differentiation, motility and invasion. PLoS Pathog.

[38] Keeley A, Soldati D (2004). The glideosome: a molecular machine powering motility and host-cell invasion by Apicomplexa. Trends Cell Biol.

[39] Moon RW, Taylor CJ, Bex C, Schepers R, Goulding D, Janse CJ, Waters AP, Baker DA, Billker O (2009). A cyclic GMP signalling module that regulates gliding motility in a malaria parasite. PLoS Pathog.

[40] Wang X, Qian P, Cui H, Yao L, Yuan J (2020). A protein palmitoylation cascade regulates microtubule cytoskeleton integrity in Plasmodium. EMBO J.

[41] Schrevel J, Asfaux-Foucher G, Hopkins JM, Robert V, Bourgouin C, Prensier G, Bannister LH (2008). Vesicle trafficking during sporozoite development in Plasmodium berghei: ultrastructural evidence for a novel trafficking mechanism. Parasitology.

[42] Sinden RE (1999). Plasmodium differentiation in the mosquito. Parassitologia.

[43] Bannister LH, Hopkins JM, Fowler RE, Krishna S, Mitchell GH (2000). Ultrastructure of rhoptry development in Plasmodium falciparum erythrocytic schizonts. Parasitology.

[44] Ouologuem DT, Roos DS (2014). Dynamics of the Toxoplasma gondii inner membrane complex. J Cell Sci.

[45] Carter V, Shimizu S, Arai M, Dessens JT (2008). PbSR is synthesized in macrogametocytes and involved in formation of the malaria crystalloids. Mol Microbiol.

[46] Saeed S, Tremp AZ, Dessens JT (2015). Biogenesis of the crystalloid organelle in Plasmodium involves microtubule-dependent vesicle transport and assembly. Int J Parasitol.

[47] Braks JA, Mair GR, Franke-Fayard B, Janse CJ, Waters AP (2008). A conserved U-rich RNA region implicated in regulation of translation in Plasmodium female gametocytes. Nucleic Acids Res.

[48] Tremp AZ, Saeed S, Sharma V, Lasonder E, Dessens JT (2020). Plasmodium berghei LAPs form an extended protein complex that facilitates crystalloid targeting and biogenesis. J Proteomics.

[49] Wu HY, Liu MS, Lin TP, Cheng YS (2011). Structural and functional assays of AtTLP18.3 identify its novel acid phosphatase activity in thylakoid lumen. Plant Physiol.

[50] Pradel G, Hayton K, Aravind L, Iyer LM, Abrahamsen MS, Bonawitz A, Mejia C, Templeton TJ (2004). A multidomain adhesion protein family expressed in Plasmodium falciparum is essential for transmission to the mosquito. J Exp Med.

[51] Saeed S, Tremp AZ, Dessens JT (2018). The Plasmodium LAP complex affects crystalloid biogenesis and oocyst cell division. Int J Parasitol.

[52] Tremp AZ, Sharma V, Carter V, Lasonder E, Dessens JT (2017). LCCL protein complex formation in Plasmodium is critically dependent on LAP1. Mol Biochem Parasitol.

[53] Santos JM, Duarte N, Kehrer J, Ramesar J, Avramut MC, Koster AJ, Dessens JT, Frischknecht F, Chevalley-Maurel S, Janse CJ (2016). Maternally supplied S-acyl-transferase is required for crystalloid organelle formation and transmission of the malaria parasite. Proc Natl Acad Sci USA.

[54] Sinden RE, Butcher GA, Billker O, Fleck SL (1996). Regulation of infectivity of Plasmodium to the mosquito vector. Adv Parasitol.

[55] Janse CJ, Franke-Fayard B, Mair GR, Ramesar J, Thiel C, Engelmann S, Matuschewski K, van Gemert GJV, Sauerwein RW, Waters AP (2006). High efficiency transfection of *Plasmodium berghei* facilitates novel selection procedures. Mol Biochem Parasitol.

[56] Burda PC, Roelli MA, Schaffner M, Khan SM, Janse CJ, Heussler VT (2015). A Plasmodium phospholipase is involved in disruption of the liver stage parasitophorous vacuole membrane. PLoS Pathog.

