# Supplementary material for: Identification of genes required for Plasmodium gametocyte-to-sporozoite development in the mosquito vector
Source: Cell Host Microbe. Author manuscript; Available in PMC 2025 Sep 9. (PMC7618085; doi:10.1016/j.chom.2023.08.010)
Supplement: Supplementary Material — Supplemental information can be found online at https://doi.org/10.1016/j.chom.2023.08.010. [file EMS208536-supplement-Supplementary_Material.zip › 1-s2.0-S1931312823003359-mmc1.pdf]

Cell Host & Microbe, Volume *31*

Supplemental information

Identification of genes required for *Plasmodium* gametocyte-to-sporozoite development in the mosquito vector

Chiamaka Valerie Ukegbu, Ana Rita Gomes, Maria Giorgalli, Melina Campos, Alexander J. Bailey, Tanguy Rene Balthazar Besson, Oliver Billker, Dina Vlachou, and George K. Christophides

Supplementary Material

Identification of genes required for *Plasmodium* gametocyte-to-sporozoite development in the mosquito vector

Chiamaka Valerie Ukegbu, Ana Rita Gomes, Maria Giorgalli, Melina Campos, Alexander Bailey, Tanguy Rene Balthazar Besson, Oliver Billker, Dina Vlachou, George K. Christophides

SUPPLEMENTARY FIGURES

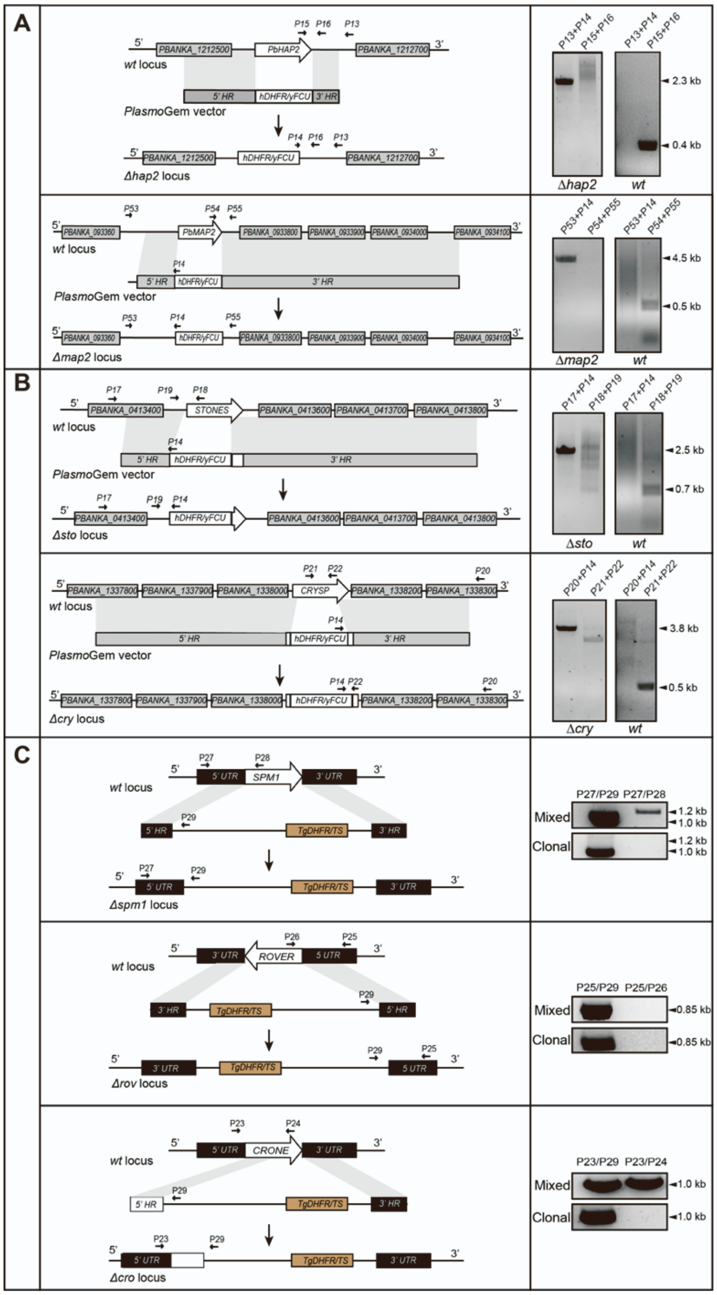

Figure S1. Generation of mutant parasites, related to Figures 2 and 4.

Schematic representation of the disruption of (**A**) *HAP2* and *MAP2* in the *1804cl1* line and (**B**) *STONES* and *CRYSP* in the *c507* line, using *Plasmo*GEM vectors carrying the *human DHFR* (*hDHFR)/yFCU* pyrimethamine resistance cassette, as well as of **(C)** *ROVER*, *CRONE* and *SPM1* in the *c507* line by using vectors that carry a modified *Toxoplasma gondii DHFR (TgDHFR)* pyrimethamine resistance expression cassette in the *c507* line**.** In each panel, the *wt* genomic locus (top), the transfected DNA fragment carrying the homologous regions (HR) and the selectable markers (middle), and the final transgenic locus after gene disruption by double crossover homologous recombination (bottom) are shown**.** Constructs are not drawn to scale. Small arrows show the primers used for the confirmation of integration of gene targeting constructs and deletion of the gene of interest using PCR as shown in the panels on the right. The *1804cl1* and *c507 wt* parental parasites were used as a control for detection of the *wt* locus.

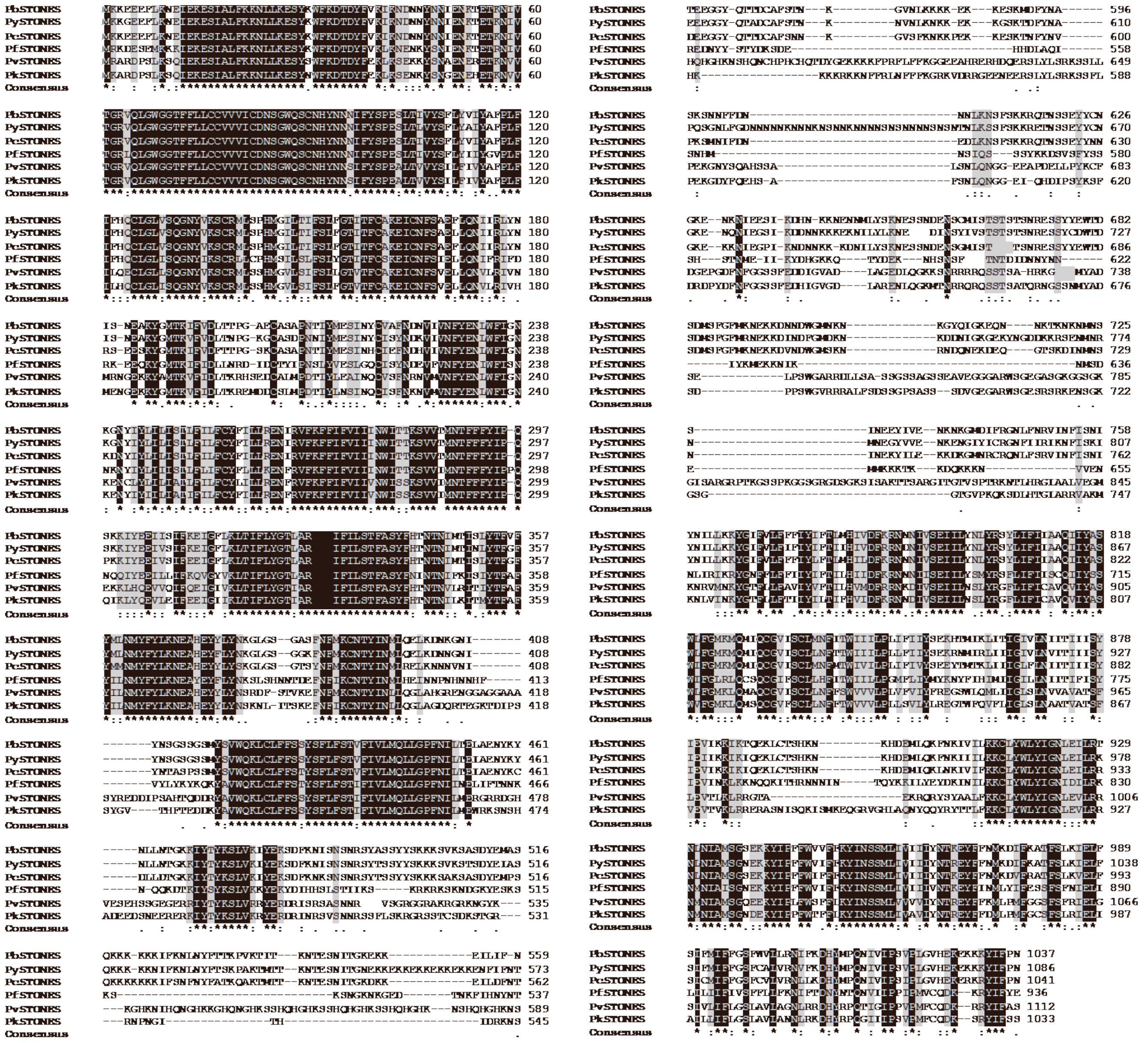

Figure S2. Multiple sequence alignment of *Plasmodium* STONES orthologs, related to Figure 4.

PBANKA_0413500 (*STONES*) encodes a 1,037 amino acid (aa) protein (122 kDa) predicted to contain 14 transmembrane domains (aa 66-86, 106-129, 141-159, 242-261, 270-295, 319-340, 347-366, 420-442, 766-784, 804-823, 830-853, 859-881, 945-966 and 986-1003). Domain analysis revealed coupled, N-terminal TOF-LisH motifs in STONES, which are thought to promote oligomerization. *Stones* orthologs are present in all sequenced *Plasmodium* species, all predicted to encode 14 transmembrane domain proteins. Protein sequence alignment shows 88%, 92%, 64%, 59% and 61% similarity to *P. yoelii* (PY17X_0416300), *P. chabaudi* (PCHAS_0414400), *P. falciparum* (PF3D7_0315700). *P. vivax* (PVX_095450) and *P. knowlesi* (PKNH_0826400) STONES, respectively. Fully conserved amino acid residues are shaded in black and similar residues in grey. In the consensus sequence, identical residues among the orthologs are represented with an asterisk and the dots and colons mark amino acid residues with weakly and strongly similar amino acid characteristics. Protein sequences were retrieved from VEuPathDB.

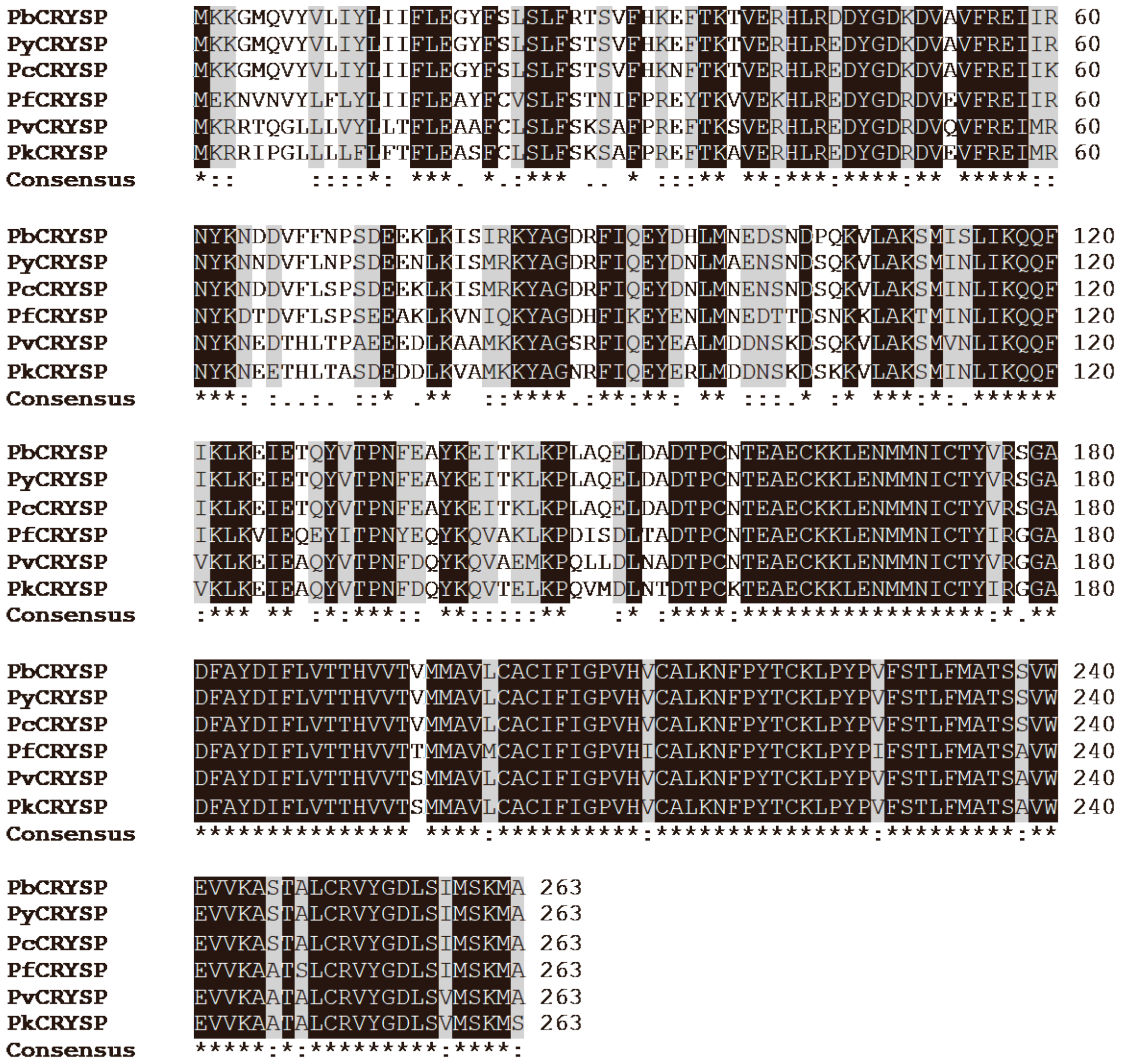

Figure S3. Multiple sequence alignment of *Plasmodium* CRYSP orthologs, related to Figure 4.

PBANKA_1338100 (CRYSP) encodes 263 amino acid (aa) protein (30.4 kDa) predicted to contain three transmembrane domains (aa 6-26, 186-212, and 224-243) but no other domains. The protein is highly conserved amongst *Plasmodium* orthologs with 97%, 87%, 87%, and 86% sequence similarity to *P. yoelii* (PY17X_1342800), *P. chabaudi* (PCHAS_1342700), *P. falciparum* (PF3D7_1322900). *P. vivax* (PVX_116725) and *P. knowlesi* (PKNH_1205900) CRYSPs, respectively. PvCRYSP and PkCRYSP are predicted to encode only two transmembrane domains. Fully conserved amino acid residues are shaded in black and similar residues in grey. In the consensus sequence, identical residues among the orthologs are represented with an asterisk and the dots and colons mark amino acid residues with weakly and strongly similar amino acid characteristics. Protein sequences were retrieved from VEuPathDB.

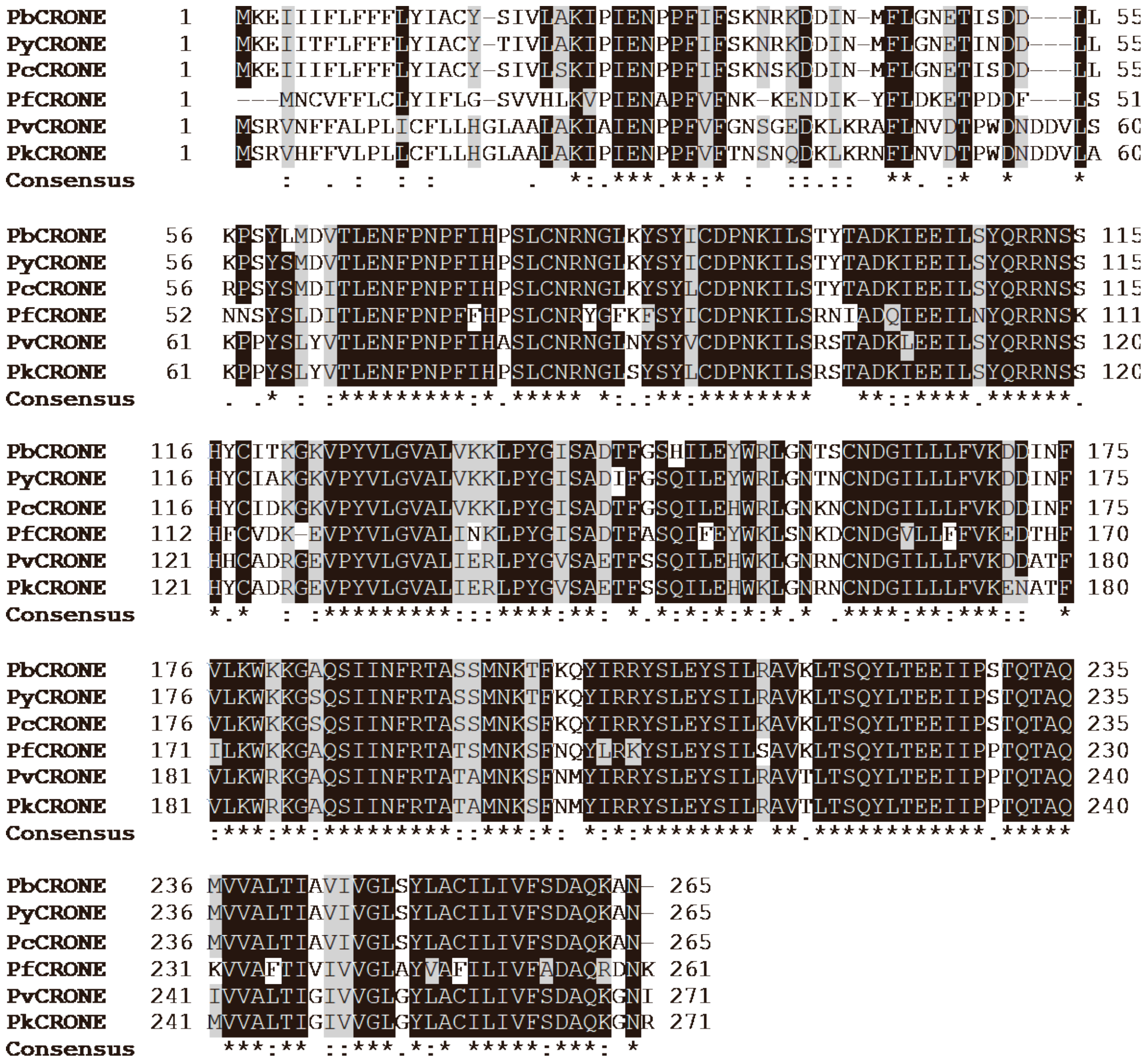

Figure S4. Multiple sequence alignment of *Plasmodium* CRONE orthologs, related to Figure 4.

PBANKA_0720900 (CRONE) encodes a 265 amino acid (aa) protein (30 kDa) with an N-terminal signal peptide (aa 1-23), a C-terminal single-pass type I transmembrane domain (aa 236-258) and a TPM domain (PFAM04536; aa 130-172). This protein architecture is predicted for all *Plasmodium* orthologs. The TPM domain, named after its founding proteins TLP18.3, Psb32 and MOLO-1, the first two of *Arabidopsis thaliana* and the third of *C. elegans*, respectively (Sirpiö et al., 2007, Wegener et al., 2011, Wu et al., 2011, Boulin et al., 2012), despite a strong structural conservation exhibits a very low sequence conservation (Eletsky et al., 2012). Nevertheless, CRONE shows high sequence similarity with its *Plasmodium* orthologs ranging from 97% with *P. yoelii* (PY17X_0720900) and 97% with *P. chabaudi* (PCHAS_0729900) to 77%, 78% and 80% with *P. falciparum* (PF3D7_0418800), *P. vivax* (PVX_090030) and *P. knowlesi* (PKNH_0511500) CRONE, respectively. Fully conserved amino acid residues are shaded in black and similar residues in grey. In the consensus sequence, identical residues among the orthologs are represented with an asterisk and the dots and colons mark amino acid residues with weakly and strongly similar amino acid characteristics. Protein sequences were retrieved from VEuPathDB.

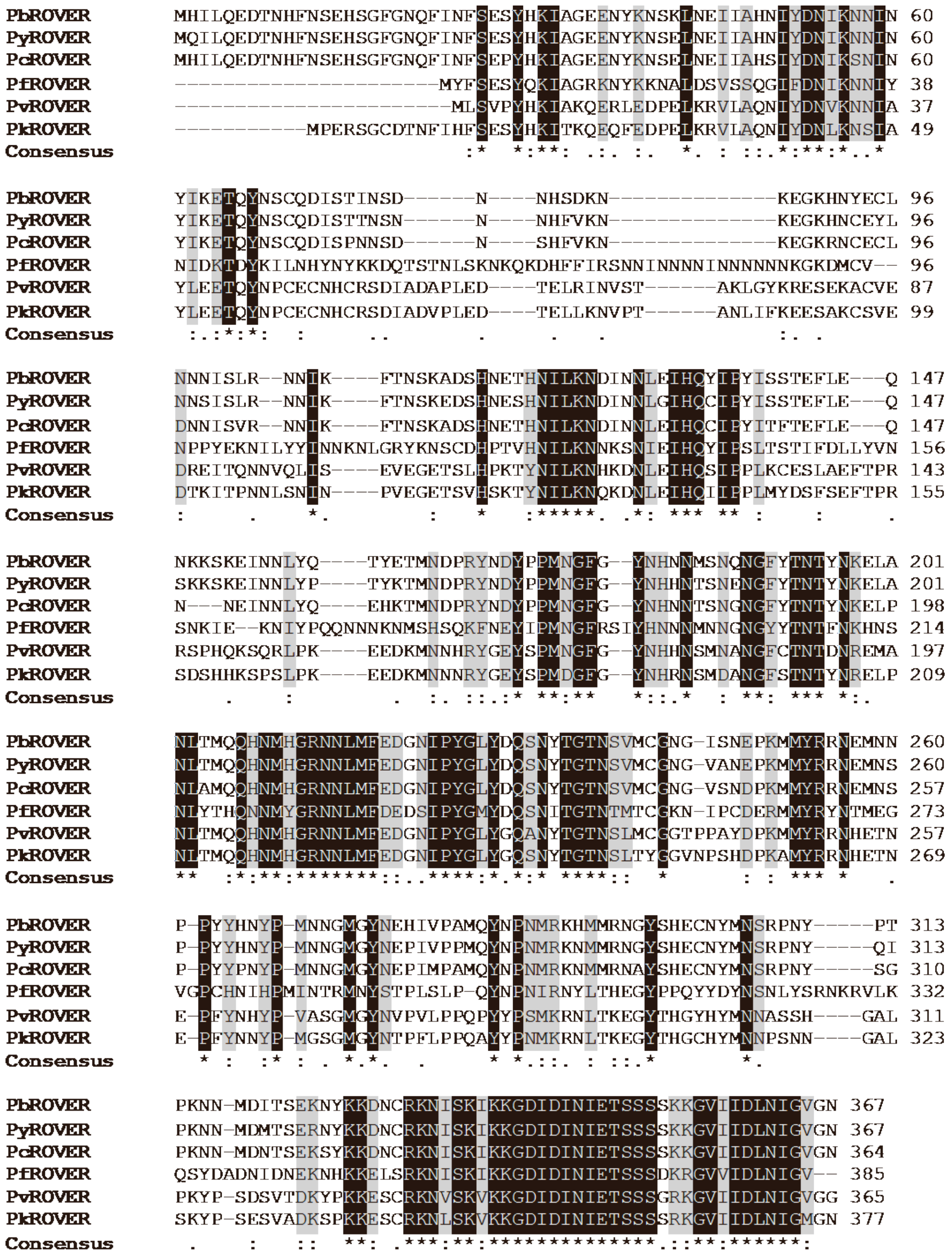

Figure S5. Multiple sequence alignment of *Plasmodium* ROVER orthologs, related to Figure 4.

PBANKA_1353800 (*ROVER*) encodes a 367 amino acid protein (43 kDa) with no signal peptide, transmembrane domain, or any other functional domain. Multiple sequence showed that PbROVER show very high sequence similarity with its *P. yoelii* (PY17X_1359200) and *P. chabaudi* (PCHAS_1358400) orthologs of 93% and 91%, respectively. Lower sequence similarity of 48%, 54% and 58% is seen with its *P. falciparum* (PF3D7_1340400), *P. vivax* (PVX_082937) and *P. knowlesi* (PKNH_1261000) orthologs, respectively. Identical amino acid residues are shaded in black and similar residues in grey. In the consensus sequence, identical residues among the orthologs are shown with an asterisk, and dots and colons mark residues with weakly and strongly similar characteristics. Protein sequences were retrieved from VEuPathDB.

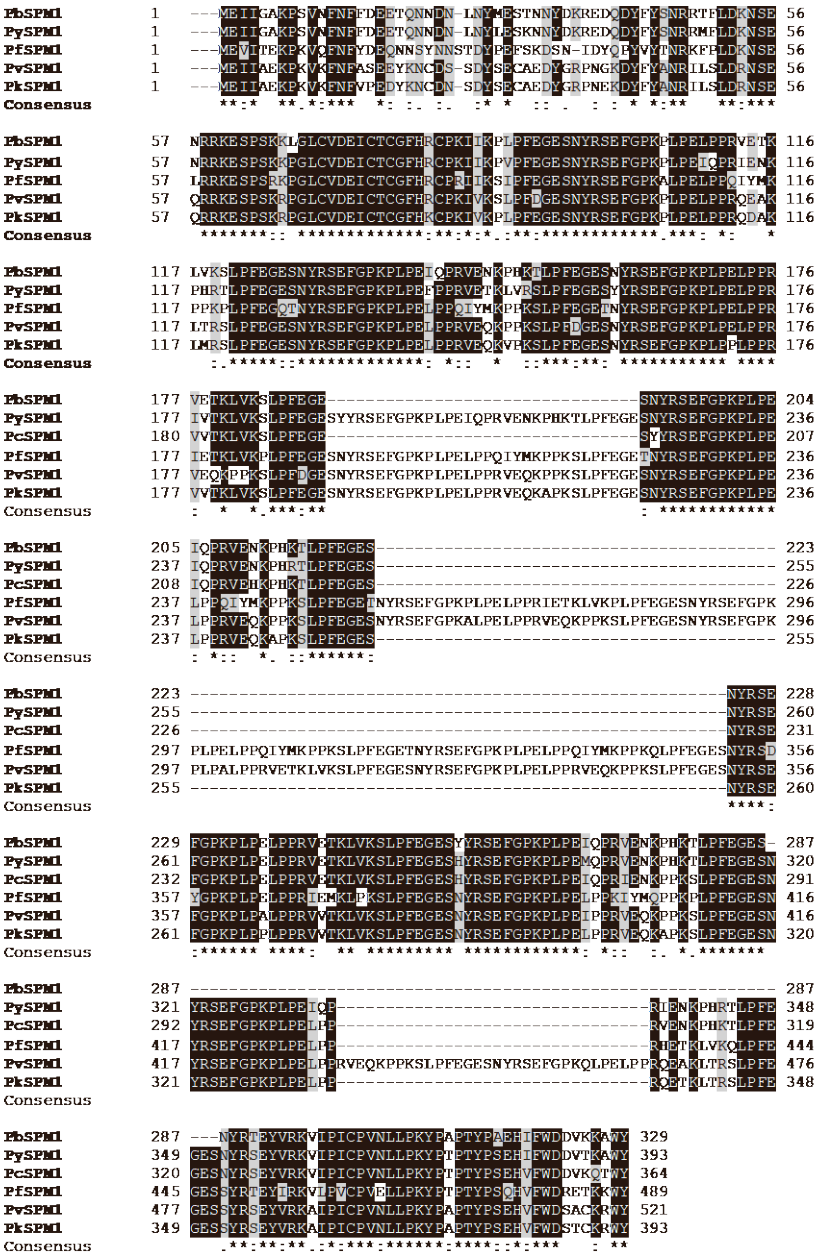

Figure S6. Multiple sequence alignment of *Plasmodium* SPM1 orthologs, related to Figure 4.

PBANKA_0810700 encodes a 329 aa (38 kDa) putative subpellicular microtubule protein, SPM1, predicted to contain a microtubule associated protein 6 (MAP6) domain (IPR024963) in aa 69-286 and no signal peptide or transmembrane domain. Subpellicular microtubules are a basic scaffold that consists of a row of microtubules that run under the pellicle and determine the rigid cell shape of several protists. They originate from the apical polar ring, and their interaction with the cytosolic face of the pellicle is mediated by the microtubule associated proteins (MAPs), including SPM1 and its paralog SPM2. SPM1 protein comparisons with other *Plasmodium* SPM1 orthologs reveals a similarity of 78%, 84%, 55%, 56% and 72% with *P. yoelii* (PY17X_0814000), *P. chabaudi* (PCHAS_0811000), *P. falciparum* (PF3D7_0909500). *P. vivax* (PVX_098915) and *P. knowlesi* (PKNH_PKNH_0707500) SPM1 orthologs, respectively*.* The MAP6 domain of PbSPM1 is made up of seven 26 aa repeats that are conserved within *Plasmodium* orthologs, ranging in number from 8 in PySPM1 to 13 in PvSPM1. Fully conserved aa residues are shaded in black and similar residues in grey. In the consensus sequence, identical residues among the orthologs are represented with an asterisk, while dots and colons mark residues with weakly and strongly similar amino acid characteristics, respectively. Protein sequences were retrieved from VEuPathDB.

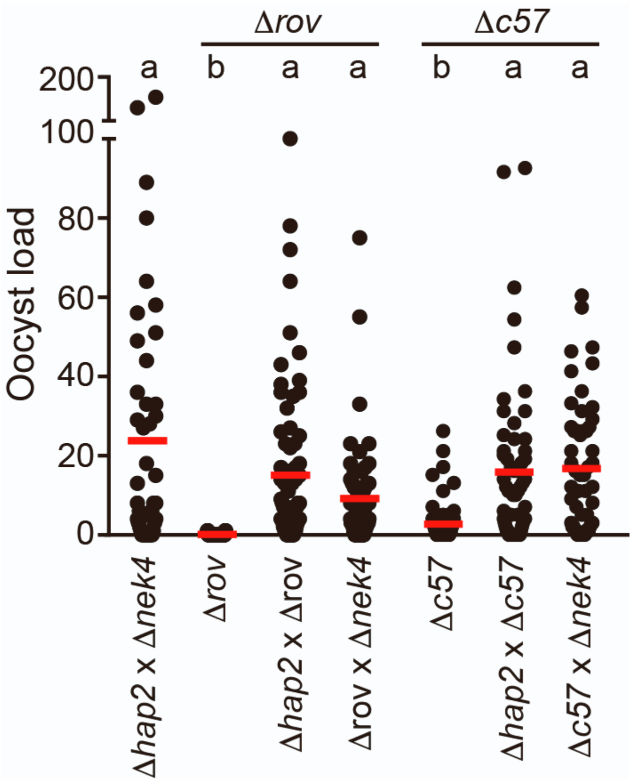

Figure S7. Genetic complementation assays of *ROVER* and *PIMMS57*, related to Figure 4.

Oocyst distribution in the midguts of *A. coluzzii* mosquitoes at day 9 pbf, following genetic crossing of *Δrov* or *Δc57* with either *Δnek4* or *Δhap2* lines, respectively. Infections of the *c507 wt* line and a genetic cross between *Δnek4* and *Δhap2* were used as controls. Two biological replicates were performed and statistical analysis was performed using a Mann–Whitney U-test. Letters (a, b and c) above each infection indicate the results from multiple comparisons: no difference was detected between infections annotated with the same letter, while a statistically significant difference ($p < 0.0001$) was detected between infections annotated with difference letters. The mean is shown with a red line.

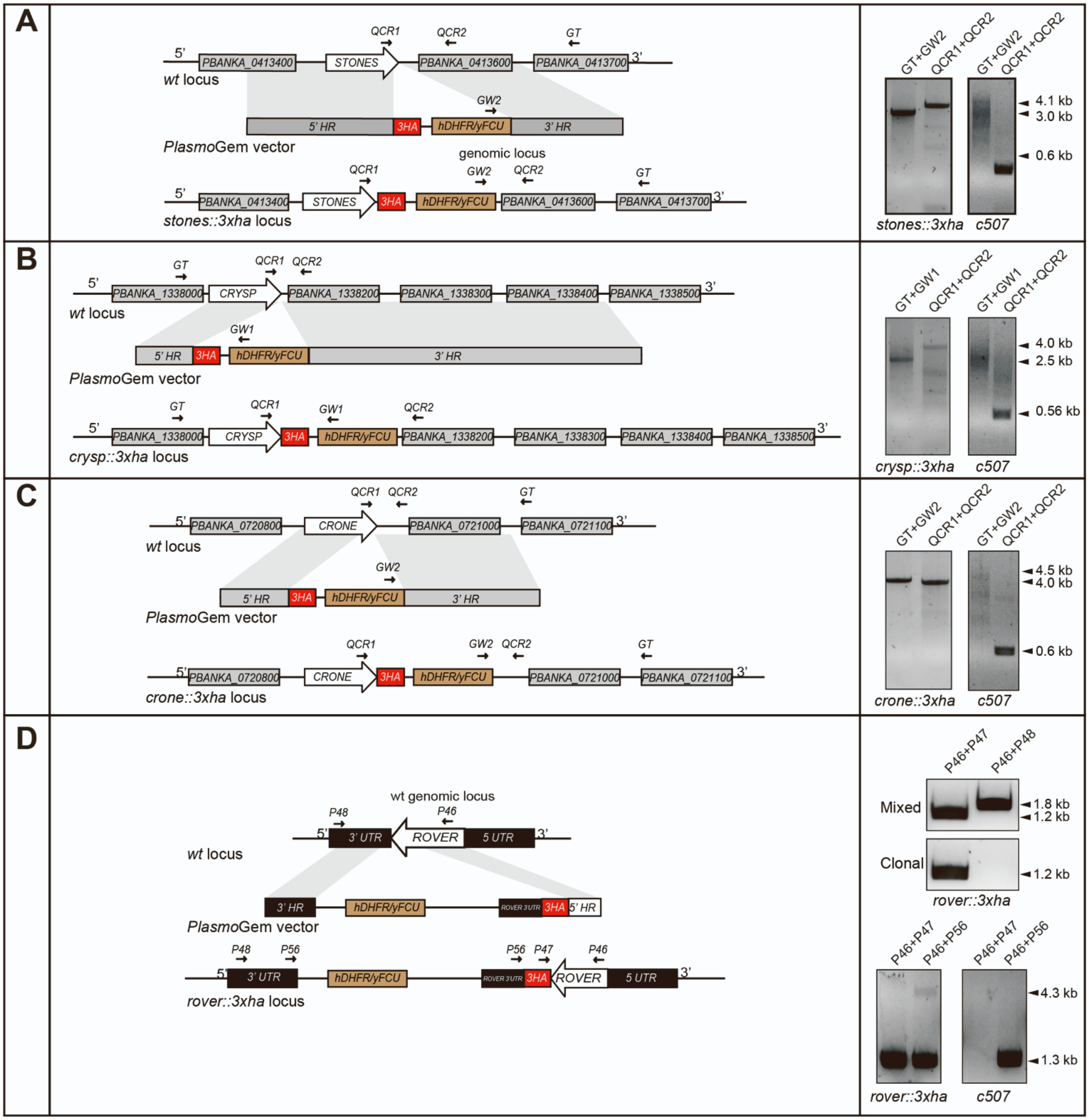

Figure S8. Generation of gene tagged parasites, related to Figure 5.

Schematic representation of the 3XHA tagging of (**A**) *STONES*, (**B**) *CRYSP* (**C**) *CRONE* and (**D**) *ROVER* in the *c507* line by using PlasmoGEM gene tagging vectors that carry the *hDHFR)/yFCU* selectable marker. In each panel, the *wt* genomic locus (top), the plasmid carrying the homologous regions (HR), the transfected DNA fragment carrying the tagging cassettes and the selectable markers (middle), and the final transgenic locus after double crossover homologous recombination (bottom) are shown. Constructs are not drawn to scale. Small arrows show the primers used for the confirmation of integration of gene targeting constructs and deletion of the gene of interest using diagnostic PCR reactions as shown in the panels on the right.

SUPPLEMENTARY TABLES

Table S3. Oocyst counts of mutant parasites and *c507* controls in *A. coluzzii*, related to Figure 4.

Parasite	Experiment	No of midguts	Prevalence (%)	Arithmetic mean	Median	Parasite Range	P value
c507	Pooled	61	82	22.1	13	0-176	
Δsto		67	0	0	0	0	<0.0001
c507		80	86	24.6	14	0-127	
Δcry		76	93	40.1	21	0-238	0.0518
c507		99	89	70.9	47	0-400	
Δcro		99	87	56.1	33	0-277	0.4245
c507		120	78	28.4	9	0-203	
Δrov		104	5	0.05	0	0-1	<0.0001
c507		106	85	39.3	32	0-176	
Δspm1		105	88	27.5	15	0-136	0.0535
Δsto	R1	32	0	0	0	0	<0.0001
c507		26	81	28.3	16	0-120	
Δcry		26	88	58.1	41	0-238	0.0579
c507		34	94	122.0	95	0-400	
Δcro		34	94	91.9	75	0-277	0.1813
c507		40	85	29.7	6	0-203	
Δrov		33	9	0.1	0	0-1	<0.0001
c507		34	74	34.8	16	0-176	
Δspm1		35	91	26.0	18	0-103	0.9166
Δsto	R2	35	0	0	0	0	<0.0001
c507		28	93	24.8	17	0-127	
Δcry		25	96	26.9	16	0-208	0.5505
c507		35	80	29.3	9	0-218	
Δcro		35	74	25.1	11	0-133	0.9930
c507		35	60	26.0	3	0-166	
Δrov		35	0	0	0	0	<0.0001
c507		32	94	48.7	44	0-149	
Δspm1		30	90	27.9	18	0-118	0.0422
c507	R3	26	85	20.6	9	0-110	
Δcry		25	96	34.6	24	0-105	0.0818
c507		45	84	29.2	15	0-137	
Δcro		30	93	51.7	33	0-165	0.8862
c507		40	88	35.7	29	0-158	
Δrov		36	6	0.1	0	0-1	<0.0001
c507		30	93	61.5	44	0-276	
Δspm1		40	83	28.7	8	0-136	0.1883
c507		40	88	35.7	29	0-158	

Table S4. Oocyst counts of mutant parasites and *c507* controls in *A. coluzzii*, related to Figure 4.

Genetic crosses	Experiment	No of midguts	Prevalence (%)	Arithmetic mean	Median	Parasite Range	P value
Δhap2 x Δnek4	pool	47	79	23.7	11.8	0-154	
Δrov		55	11	0.1	0.05	0-1	<0.0001
Δhap2 x Δrov		76	84	15	7.5	0-38	0.59
Δnek4 x Δrov		58	74	9.1	4.6	0-75	0.59
Δc57		69	49	2.6	1.3	0-26	<0.0001
Δhap2 x Δc57		58	79	15.7	7.9	0-92	0.64
Δ nek4 x Δc57		53	87	16.6	8.3	0-60	0.98

Table S5. Midgut and salivary glands sporozoites and mosquito-to-mouse transmission, related to Figure 4.

	Midgut sporozoites				Salivary gland sporozoites				
Parasite	Reps	Mean	SEM	P	Reps	Mean	SEM	P	Bite-back
c507	2	3,567	259		2	1,843	83		6/6(3/3;3/3)
Δsto	2	10	7	0.011	2	67	0	0.004	0/6(0/3;0/3)
c507	3	6,036	180		3	3,484	480		6/6(2/2;2/2;2/2)
Δcry	3	6,391	745	0.99	3	0	0	0.004	0/6(0/2;0/2;0/2)
c507	3	10,694	902		3	7,587	925		9/9(3/3;3/3;3/3)
Δcro	3	0	0	0.001	3	0	0	0.003	0/9(0/3;0/3;0/3)
c507	3	3,556	970		3	4,344	1,086		9/9(3/3;3/3;3/3)
Δrov	3	61	11	0.042	3	16	9	0.031	0/9(0/3;0/3;0/3)
c507	3	2,268	488		3	4,668	458		9/9(3/3;3/3;3/3)
Δspm1	3	1,121	206	0.152	3	2,007	28	0.009	9/9(3/3;3/3;3/3)

For each biological replicate, sporozoite numbers was determined from 25-30 homogenized mosquito midguts or salivary glands at 15 and 21 dpbf respectively. Infectivity of sporozoites was assessed by infected mosquito bite back experiments with at least 30 mosquitoes on C57/BL6 mice at 21 dpbf. Following this, parasitemia was monitored until 14 days post mosquito bite. *P* values were calculated using the unpaired Student's t-test. SEM represents standard error of mean.

Table S6. Invasion assay in *CTL4* knockdown *A. coluzzii*, related to Figure 5.

Parasite	Experiment	No of midguts	Prevalence (%)	Arithmetic mean	Median	Parasite range	P value
c507	Pool	60	83	162.3	186	0-389	
Δsto		77	22	0.7	0	0-11	<0.0001
c507		80	78	56.0	19	0-558	
Δrov		89	24	3.2	0	0-69	<0.0001
c507	R1	34	82	161.8	178	0-370	
Δsto		43	21	0.6	0	0-7	<0.0001
c507		15	80	52.3	19	0-193	
Δrov		23	4	0.1	0	0-2	<0.0001
c507	R2	26	85	163.0	188	0-389	
Δsto		34	24	0.9	0	0-11	<0.0001
c507		30	73	65.2	19	0-533	
Δrov		30	3	0.1	0	0	<0.0001
c507	R3	35	83	49.7	13	0-558	
Δrov		36	53	7.8	1	0-69	0.0006

Numbers of melanised parasites detected in the midguts of *c507* infected *CTL4* kd *A. coluzzii* mosquitoes at 7days post feeding. The P value was calculated using the Mann-Whitney U test. Data from pooled and independent biological replicates are presented.

Table S7. Sporozoite numbers and mosquito-to-mouse transmission after ookinete injection, related to Figure 5.

	Salivary gland sporozoites		Infectivity to mice
Parasite	Mean	SEM	
c507	3,353(3,458; 3,248)	74	4/4(2/2;2/2)
Δsto	3,093(3,199; 2,987)	75	4/4(2/2;2/2)
c507	3,258(3,036; 1,888; 4,850)	704	6/6(2/2;2/2;2/2)
Δrov	2,439(2,960; 958; 3,400)	614	6/6(2/2;2/2;2/2)

Mean salivary gland sporozoites at 21 days post *A. coluzzii* haemocoel inoculation with ookinetes, obtained from 6 biological replicates. Data from independent biological replicates are presented in brackets. Infectivity of sporozoites was assessed by infected mosquito bite back experiments of C57/BL6 mice at day 21 post haemocoel inoculation. Parasitaemia was monitored for 14 days post mosquito bite. SEM represents the standard error of the mean.

Table S8. Barcode and indexing primers, related to STAR Methods.

Primer name	Sequence (5′ to 3′)	Primer description
BC_STM Primer 91_Illumina	TCGGCATTCCTGCTGAACCGCTCTTCCGATCT*GTAATTCGTGCGCGTCAG*	Barcode upstream
BC_STM Primer 97_Illumina	ACACTCTTTCCCTACACGACGCTCTTCCGATCT*CCTTCAATTTCGATGGGTAC*	Barcode downstream
PE 1.0 Primer_Illumina PCR	**AATGATACGGCGACCACCGAGATCTACAC**TCTTTCCCTACACGACGCTCTTCCGATC*T	Index upstream
iPCRindex1	**CAAGCAGAAGACGGCATACGAGAT**TGCTAATCACTGAGATCGGTCTCGGCATTCCTGCTGA ACCGCTCTTCCGATC*T	Index 1 downstream
iPCRindex2	**CAAGCAGAAGACGGCATACGAGAT**TAGGGGATTCGAGATCGGTCTCGGCATTCCTGCTGA ACCGCTCTTCCGATC*T	Index 2 downstream
iPCRindex3	**CAAGCAGAAGACGGCATACGAGAT**AGTTTCCCAGGGAGATCGGTCTCGGCATTCCTGCTGA ACCGCTCTTCCGATC*T	Index 3 downstream
iPCRindex4	**CAAGCAGAAGACGGCATACGAGAT**CCTGGGAGGTAGAGATCGGTCTCGGCATTCCTGCTG AACCGCTCTTCCGATC*T	Index 4 downstream
iPCRindex5	**CAAGCAGAAGACGGCATACGAGAT**ATACCACAAATGAGATCGGTCTCGGCATTCCTGCTGA ACCGCTCTTCCGATC*T	Index 5 downstream
iPCRindex6	**CAAGCAGAAGACGGCATACGAGAT**GATCTCTCGGGGAGATCGGTCTCGGCATTCCTGCTGA ACCGCTCTTCCGATC*T	Index 6 downstream
iPCRindex7	**CAAGCAGAAGACGGCATACGAGAT**ACCCTATACTCGAGATCGGTCTCGGCATTCCTGCTGA ACCGCTCTTCCGATC*T	Index 7 downstream
iPCRindex8	**CAAGCAGAAGACGGCATACGAGAT**CTCAATTAAGAGAGATCGGTCTCGGCATTCCTGCTGA ACCGCTCTTCCGATC*T	Index 8 downstream
iPCRindex9	**CAAGCAGAAGACGGCATACGAGAT**CGACAGAACGTGAGATCGGTCTCGGCATTCCTGCTGA ACCGCTCTTCCGATC*T	Index 9 downstream
iPCRindex10	**CAAGCAGAAGACGGCATACGAGAT**TCGCCATTATGGAGATCGGTCTCGGCATTCCTGCTGA ACCGCTCTTCCGATC*T	Index 10 downstream

Primers used for the addition by overlap PCR of Illumina adaptors and indices to the samples of pools of transgenic parasites collected. Barcode binding sites are italicized; Illumina adaptors are in bold; i7 indices are underlined. All primers are listed in a 5′ to 3′ direction. The star (*) indicates the site of the phosphorothioate bond modification for preventing nuclease-induced degradation of the PCR product.

Table S9. Primers for generation of transgenic parasites and protein expression, related to STAR Methods

Primer name	Sequence (5′ to 3′)	Description
P1 F	TTGGGCCCGTATATTGCATGCTATTCAATTGTATTG	CRONE disruption upstream target ApaI
P2 R	CCAAGCTTGAACTTTTCCTTTGGTTATGCAATAATG	CRONE disruption upstream target HindIII
P3 F	TGAATTCGATGATCAACCAAATTTTTTGTAGAG	CRONE disruption downstream target EcoRI
P4 R	TTGGATCCGCGCATAAGTGCACACTTGTTATATAG	CRONE disruption downstream target BamHI
P5 F	TTGGGCCCGTGCACTAAGATTTGCGCATTTTATCAAATG	ROVER disruption upstream target ApaI
P6 R	CCAAGCTTCAAAAGATTATAAAATTAATAAGTC	ROVER disruption upstream target HindIII
P7 F	TGAATTCGAATATTAAAAAATTAGCTATAATTAG	ROVER disruption downstream target EcoRI
P8 R	TTGGATCCCACACATATGTGTGTATATCCAATATTC	ROVER disruption downstream target BamHI
P9 F	TTGGGCCCGTTGCTGTATCTACACATTTTAACCTG	SPM1 disruption upstream target ApaI
P10 R	CCAAGCTTGGAAAGTCGTATATTCTTTTCGTTTTATG	SPM1 disruption upstream target HindIII
P11 F	TGAATTCGTGCAGAGATGTATGAGTACATAGATGT	SPM1 disruption downstream target EcoRI
P12 R	TTGGATCCGCATAATTGGATATATATACTTGG	SPM1 disruption downstream target BamHI
PlasmoGEM HAP2 GT R (P13)	TGCCCCATTTATTTTTGTCCT	Diagnostic primer WT and KO
PlasmoGEM GW2 (P14)	CTTTGGTGACAGATACTAC	Diagnostic primer WT
PlasmoGEM HAP2 QCR1 (P15)	TGCAGATACATCTCCGTCAGGT	Diagnostic primer WT
PlasmoGEM HAP2 QCR2 (P16)	TGTTGTGTTTCCTCCATCCA	Diagnostic primer KO/c507
PlasmoGEM STONES GT F (P17)	TGGACCCGAGGATGTCACATGGG	Diagnostic primer WT and KO/c507
PlasmoGEM STONES QCR1 (P18)	TGCGCATTCTGCTCCTGGGG	Diagnostic primer WT and KO/c507
PlasmoGEM STONES QCR2 (P19)	CACAGTTTGTGCAGAGAAT	Diagnostic primer WT/c507
PlasmoGEM CRYSP GT R (P20)	ATTGCACGTTGAATATGCCA	Diagnostic primer WT and KO/c507
PlasmoGEM CRYSP QCR1 (P21)	CGAGGCGGAATGCAAAAAGCT	Diagnostic primer WT/c507
PlasmoGEM CRYSP QCR2 (P22)	CCACTTCCCAAACAGAGGATGTAGCC	Diagnostic primer KO/c507
CRONE INT F (P23)	GCATTGTTTATATACGCTTCATAAGTTTTG	Diagnostic primer WT and KO/c507
CRONE WT R (P24)	CATAAGGCAATTTTTTAACTAAGGCCAC	Diagnostic primer WT/c507
ROVER INT F (P25)	GCTTATGTTATTATTTAATATCCCCTT	Diagnostic primer WT and KO/c507
ROVER WT R (P26)	CCATTTCCACACATAACTGAATTTGTTCC	Diagnostic primer WT/c507
SPM1 INT F (P27)	GGTTTATGGACAAAAAAACAAATTAGCAG	Diagnostic primer WT and KO/c507
SPM1 WT R (P28)	CTTCATCAAAAAAATTAAAATTTACAC	Diagnostic primer WT/c507
TgDHFR 5'UTR R (P29)	GATGTGTTATGTGATTAATTCATACAC	Diagnostic primer KO/c507
ROVERHA 5’ F (P30)	GATTACGCCAAGCTTGGGCCCGAATGACCCTAGATACAATGATTATCCACCTATGAACGGATTTGG	ROVER tagging upstream target ApaI
ROVERHA 5’ R (P31)	CTAAGCATAGTCTGGAACGTCATAAGGGTATGCGTAATCTGGCACGTCGTATGGATATGCA<br>TAATCTGGTACATCGTATGGGTAATTTCCTACACCAATATTTAAGTCAATTATAACTCC	ROVER tagging upstream target
ROVERHA 3’ UTR F (P32)	GACGTTCCAGACTATGCTTAGGCAAAAGATTATAAAATTAATAAGTCAAGAGTTTG	ROVER 3’UTR
ROVERHA 3’ UTR R (P33)	CTAGAGCGGCCGCCACCGCGGGTGCACTAAGATTTGCGCATTTTATCAAATGTTTACC	ROVER 3’UTR SacII
ROVERHA 3’ F (P34)	CTTCAATTTCGGGTACCCTCGAGGCAAAAGATTATAAAATTAATAAGTCAAGAGTTTG	ROVER tagging downstream target XhoI
ROVERHA 3’ R (P35)	GAATTCGCGGCCGCCCCGGGGGCGATAACAAGAATGATGATAATAACGATCCAGGAG	ROVER tagging downstream target XmaI
PlasmoGEM STONESHA GT R (P36)	TGCCAAACTGTCAGAGGCATCA	Diagnostic primer tag
PlasmoGEM STONESHA QCR1 (P37)	AGACCATTACATGCCGCAAA	Diagnostic primer tag

PlasmoGEM STONESHA QCR2 (P38)	ACAAAACGGCAACTGACTTTGC	Diagnostic primer tag
PlasmoGEM CRYSPHA GT F (P39)	TCCCCACCTTTTCCTTCCCT	Diagnostic primer tag
PlasmoGEM GW1 (P40)	CATACTAGCCATTTTATGTG	Diagnostic primer tag
PlasmoGEM CRYSPHA QCR1 (P41)	GGCTACATCCTCTGTTTGGGAAGTGG	Diagnostic primer tag
PlasmoGEM CRYSPHA QCR2 (P42)	ACCAGCACTCGGAATTGCCA	Diagnostic primer tag
PlasmoGEM CRONEHA GT F (P43)	AGTGGGGGAAGATGGGGAGGA	Diagnostic primer tag
PlasmoGEM CRONEHA QCR1 (P44)	CCGTCCACACAAACGGCCCA	Diagnostic primer tag
PlasmoGEM CRONEHA QCR2 (P45)	TGTTTGCTTTTATTAAGAATGGGCT	Diagnostic primer tag
ROVERHA INT F (P46)	GCAGATTCACATAACGAAACTCATAATATATTG	Diagnostic primer tag
HA INT R (P47)	GTATGCGTAATCTGGCACGTCGTATG	Diagnostic primer tag
ROVERHA WT R (P48)	GATCTATTATTAAACCATATTCATTTAG	Diagnostic primer tag
CRONE GA F (P49)	AGCTCCGTCGACAAGCTTGCGGCCAAGATCCCCATCGAGAACCCCCC	STONE optimized expression primer
CRONE GA R (P50)	TGGTGGTGGTGCTCGAGTGCGGCCGCCTGAGCGGTCTGGGTGGAGG	STONE optimized expression primer
CTL4F (P51)	TAATACGACTCACTATAGGGTGGTTTGATGCCGTGTCCT	
CTL4R (P52)	TAATACGACTCACTATAGGGAATAAATTGTCTCGGTTCATCATC	
PlasmoGEM MAP2 GT F (P53)	ACCATGAGTGCATGCATAGGA	Diagnostic primer WT and KO
PlasmoGEM MAP2 QCR1 (P54)	ACGAATCACAATTGACCAGGCT	Diagnostic primer WT
PlasmoGEM MAP2 QCR2 (P55)	TGCGTGTTTGTGAAACTAATGAGGCA	Diagnostic primer KO/c507
ROVERHA WT AND HA R (P56)	CAATATGCCTACATGCTCAGTGTAATATACAAACTC	Diagnostic primer KO/c507

Where appropriate, target restriction sites are shown as underlined italics. The appropriate restriction enzyme is presented in the description column. F, forward; R, reverse; INT, integration; WT, wild-type; KO, knockout; UTR, untranslated region; GA, Gibson assembly. All primers are listed in a 5′ to 3′ direction.
